# Morphology, molecular genetics, and acoustics reveal two new species of the genus *Leptobrachella* from northwestern Guizhou Province, China (Anura, Megophryidae)

**DOI:** 10.3897/zookeys.848.29181

**Published:** 2019-05-20

**Authors:** Jian Wang, Yu-Long Li, Yao Li, Hong-Hui Chen, Ya-Jun Zeng, Jing-Min Shen, Ying-Yong Wang

**Affiliations:** 1 State Key Laboratory of Biocontrol / The Museum of Biology, School of Life Sciences, Sun Yat-sen University, Guangzhou 510275, China Sun Yat-sen University Guangzhou China; 2 Guizhou Academy of Forestry, Guizhou 550005, China Guizhou Academy of Forestry Guizhou China

**Keywords:** Acoustics, *Leptobrachellabijie* sp. nov., *L.purpuraventra* sp. nov., molecular phylogeny, morphology, taxonomy

## Abstract

Two new species of the genus *Leptobrachella* Smith, 1925, *L.bijie* J. Wang, Y.L. Li, Y. Li, H.H. Chen & Y.Y. Wang, **sp. nov.** and *L.purpuraventra* J. Wang, Y.L. Li, Y. Li, H.H. Chen & Y.Y. Wang, **sp. nov.**, were described from northwestern Guizhou Province, China based on a combination of acoustic, molecular, and morphological data. The new discoveries bring the total number of this genus to 73, with 16 congeners recorded in China, and represent the second and third species of the genus reported from Guizhou Province.

## Introduction

The Asian leaf litter toad genus *Leptobrachella* Smith, 1925 currently contains seventy-one species, widely distributed from southern China west to northeastern India and Myanmar, through mainland Indochina to peninsular Malaysia and the island of Borneo (Eto et al. 2018; Frost 2017; Nguyen et al. 2018; Rowley et al. 2016, 2017; Yang et al. 2016; Yuan et al. 2017). Currently, 14 species of this genus are known from China, i.e., *L.alpinus* from Yunnan and Guangxi provinces, *L.laui* from southern Guangdong Province including Hong Kong, *L.liui* from Fujian, Jiangxi, Guangdong, Guangxi, Hunan and Guizhou provinces, *L.mangshanensis* from southern Hunan Province, *L.oshanensis* from Gansu, Sichuan, Chongqing, Guizhou and Hubei provinces, L.cf.pelodytoides (which may be a population of *L.eos* (Ohler et al. 2011)), *L.purpura*, *L.tengchongensis*, *L.ventripuntatus* and *L.yingjiangensis* from Yunnan Province, *L.wuhuangmontis* from southern Guangxi Province, *L.yunkaiensis* from western GuangdongProvince, and *L.sungi* and *L.maoershanensis* from Guangxi Province (Hou et al. 2018; Sung et al. 2014; Wang et al. 2018; Yang et al. 2016; Yuan et al. 2017, Yang et al. 2018).

**Figure 1.**
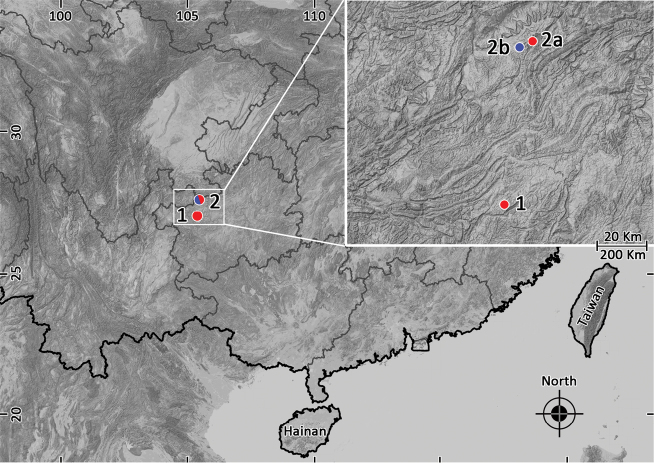
Collection localities of the two new *Leptobrachella* species: **1** Jinjiazhai Village in Wujing Nature Reserve, the type locality of *L.purpuraventra* sp. nov. **2a** Baimashan Forest Station in Zhaozishan Nature Reserve, the other collection site of *L.purpuraventra* sp. nov. **2b** Qingshan Village in Zhaozishan Nature Reserve, the type locality of *L.bijie* sp. nov.

During recent field surveys in northwestern Guizhou Province of China in 2018, a number of specimens were collected from Zhaozishan Nature Reserve and Wujing Nature Reserve in Qixingguan District of Bijie City, respectively (Figure 1), which can be morphologically assigned to the genus *Leptobrachella*, based on the following characters: (1) small or moderate size, snout-vent length not greater than 60.0 mm, (2) rounded finger tips, the presence of an elevated inner palmar tubercle not continuous to the thumb, (3) presence of macroglands on body including supra-axillary, pectoral, femoral and ventrolateral glands, (4) vomerine teeth absent, (5) tubercles on eyelids present, and (6) anterior tip of snout with whitish vertical bar (Dubois 1983; Matsui 1997, 2006; Lathrop et al. 1998; Delorme et al. 2006; Das et al. 2010). Subsequent 16S rRNA sequences from these specimens revealed that these collections represent two distinct evolving lineages. Combine of morphological characters, acoustic data, and molecular divergences; they are described herein as two new species.

## Material and methods

### Sampling

For molecular analyses, a total of 71 sequences (23 muscle tissues were sequenced and 48 sequences downloaded from GenBank) from 32 species of the genus *Leptobrachella* were used, including two undescribed species from China, i.e., the populations from Zhaozishan Nature Reserve and Wujing Nature Reserve of Guizhou Province. And four sequences were downloaded from GenBank as outgroups (see Table 1; *Pelobatessyriacus*, *P.varaldii*, Leptobrachiumcf.chapaense, and *Megophrysmajor*).

**Table 1.** Localities and voucher data for all specimens used in this study.

ID	Species	Locality	voucher No.	GenBank No.
				16S rRNA
1	*Leptobrachellapurpuraventra* sp. nov.	China: Wujing Nature Reserve, Bijie City, Guizhou	SYS a007081	MK414517
2	*Leptobrachellapurpuraventra* sp. nov.	China: Wujing Nature Reserve, Bijie City, Guizhou	SYS a007277/CIB 110003	MK414518
3	*Leptobrachellapurpuraventra* sp. nov.	China: Wujing Nature Reserve, Bijie City, Guizhou	SYS a007278	MK414519
4	*Leptobrachellapurpuraventra* sp. nov.	China: Wujing Nature Reserve, Bijie City, Guizhou	SYS a007279	MK414520
5	*Leptobrachellapurpuraventra* sp. nov.	China: Wujing Nature Reserve, Bijie City, Guizhou	SYS a007280	MK414521
6	*Leptobrachellapurpuraventra* sp. nov.	China: Wujing Nature Reserve, Bijie City, Guizhou	SYS a007282	MK414522
7	*Leptobrachellapurpuraventra* sp. nov.	China: Wujing Nature Reserve, Bijie City, Guizhou	SYS a007283	MK414523
8	*Leptobrachellapurpuraventra* sp. nov.	China: Wujing Nature Reserve, Bijie City, Guizhou	SYS a007284	MK414524
9	*Leptobrachellapurpuraventra* sp. nov.	China: Zhaozishan Nature Reserve, Bijie City, Guizhou	SYS a007300	MK414525
10	*Leptobrachellapurpuraventra* sp. nov.	China: Zhaozishan Nature Reserve, Bijie City, Guizhou	SYS a007301	MK414526
11	*Leptobrachellapurpuraventra* sp. nov.	China: Zhaozishan Nature Reserve, Bijie City, Guizhou	SYS a007302	MK414527
12	*Leptobrachellapurpuraventra* sp. nov.	China: Zhaozishan Nature Reserve, Bijie City, Guizhou	SYS a007303	MK414528
13	*Leptobrachellapurpuraventra* sp. nov.	China: Zhaozishan Nature Reserve, Bijie City, Guizhou	SYS a007304	MK414529
14	*Leptobrachellapurpuraventra* sp. nov.	China: Zhaozishan Nature Reserve, Bijie City, Guizhou	SYS a007305	MK414530
15	*Leptobrachellapurpuraventra* sp. nov.	China: Zhaozishan Nature Reserve, Bijie City, Guizhou	SYS a007306	MK414531
16	*Leptobrachellabijie* sp. nov.	China: Zhaozishan Nature Reserve, Bijie City, Guizhou	SYS a007313/CIB 110002	MK414532
17	*Leptobrachellabijie* sp. nov.	China: Zhaozishan Nature Reserve, Bijie City, Guizhou	SYS a007314	MK414533
18	*Leptobrachellabijie* sp. nov.	China: Zhaozishan Nature Reserve, Bijie City, Guizhou	SYS a007315	MK414534
19	*Leptobrachellabijie* sp. nov.	China: Zhaozishan Nature Reserve, Bijie City, Guizhou	SYS a007316	MK414535
20	*Leptobrachellabijie* sp. nov.	China: Zhaozishan Nature Reserve, Bijie City, Guizhou	SYS a007317	MK414536
21	*Leptobrachellabijie* sp. nov.	China: Zhaozishan Nature Reserve, Bijie City, Guizhou	SYS a007318	MK414537
22	*Leptobrachellabijie* sp. nov.	China: Zhaozishan Nature Reserve, Bijie City, Guizhou	SYS a007319	MK414538
23	*Leptobrachellabijie* sp. nov.	China: Zhaozishan Nature Reserve, Bijie City, Guizhou	SYS a007320	MK414539
24	* Leptobrachella aerea *	Vietnam: Quang Binh	RH60165	JN848437
25	* Leptobrachella applebyi *	Vietnam: Kon Tum	AMS R 173778	KR018108
26	* Leptobrachella applebyi *	Vietnam: Kon Tum	AMS R 173635	KU530189
27	* Leptobrachella bidoupensis *	Vietnam: Lam Dong	AMS R 173133	HQ902880
28	* Leptobrachella bidoupensis *	Vietnam: Lam Dong	NCSM 77321	HQ902883
29	* Leptobrachella bourreti *	Vietnam: Lao Cai	AMS R 177673	KR018124
30	* Leptobrachella eos *	Lao: Phongsaly	MNHN: 2004.0278	JN848450
31	* Leptobrachella firthi *	Vietnam: Kon Tum	AMS R 176524	JQ739206
32	* Leptobrachella fritinniens *	Malaysia: Borneo	KUHE55371	AB847557
33	* Leptobrachella gracilis *	Malaysia: Borneo	KUHE55624	AB847560
34	* Leptobrachella hamidi *	Malaysia: Borneo	KUHE17545	AB969286
35	* Leptobrachella heteropus *	Malaysia: Peninsula	KUHE15487	AB530453
36	* Leptobrachella isos *	Vietnam: Gia Lai	VNMN A 2015.4 / AMS R 176480	KT824769
37	* Leptobrachella laui *	China: San zhoutian, Shenzhen	SYS a002540	MH055904
38	* Leptobrachella laui *	China: Mt. Wutong, Shenzhen	SYS a003477	MH605576
39	* Leptobrachella liui *	China: Mt. Wuyi, Fujian	SYS a002478	MH605573
40	* Leptobrachella liui *	China: Mt. Huanggang, Jiangxi	SYS a001620	KM014549
41	* Leptobrachella mangshanensis *	China: Mangshan, Hunan	MSZTC201702	MG132197
42	* Leptobrachella mangshanensis *	China: Mangshan, Hunan	MSZTC201703	MG132198
43	* Leptobrachella marmorata *	Malaysia: Borneo	KUHE53227	AB969289
44	* Leptobrachella maura *	Malaysia: Borneo	SP21450	AB847559
45	* Leptobrachella macrops *	Vietnam: Phu Yen Prov.	ZMMU-A5823	MG787993
46	* Leptobrachella maoershanensis *	China: Maoershan, Guangxi	KIZ019386	KY986931
47	* Leptobrachella melica *	Cambodia: Ratanakiri	MVZ258198	HM133600
48	* Leptobrachella minima *	Thailand: Chiangmai	/	JN848369
49	* Leptobrachella nyx *	/	ROM26828	MH055818
50	* Leptobrachella oshanensis *	China: Sichuan	SYSa001830	KM014810
51	* Leptobrachella pallida *	Vietnam: Lam Dong	UNS00511	KU530190
52	* Leptobrachella picta *	Malaysia: Borneo	UNIMAS 8705	KJ831295
53	* Leptobrachella pluvialis *	Vietnam: Lao Cai	MNHN:1999.5675	JN848391
54	* Leptobrachella pururus *	China: Yingjiang, Yunnan	SYS a006530	MG520354
55	* Leptobrachella pyrrhops *	Vietnam: Lam Dong	ZMMU A-5208	KP017575
56	* Leptobrachella pyrrhops *	Vietnam: Lam Dong	ZMMU A-4873 (ABV-00213)	KP017576
57	* Leptobrachella sabahmontana *	Malaysia: Borneo	BORNEENSIS 12632	AB847551
58	* Leptobrachella tengchongensis *	China: Tengchong County, Yunnan	SYS a004596	KU589208
59	* Leptobrachella tengchongensis *	China: Tengchong County, Yunnan	SYS a004598	KU589209
60	* Leptobrachella ventripunctata *	Laos: Phongsaly	MNHN 2005.0116	JN848410
61	* Leptobrachella ventripunctata *	China: Zhushihe, Xishuangbanna, Yunnan	SYS a001768	KM014811
62	* Leptobrachella yingjiangensis *	China: Yingjiang, Yunnan	SYS a006533	MG520350
63	* Leptobrachella yunkaiensis *	China: Dawuling Forest Station, Maoming City, Guangdong	SYS a004663	MH605584
64	* Leptobrachella yunkaiensis *	China: Dawuling Forest Station, Maoming City, Guangdong	SYS a004664 / CIB107272	MH605585
65	* Leptobrachella wuhuangmontis *	China: Mt. Wuhuang, Pubei County, Guangxi	SYS a003485	MH605577
66	* Leptobrachella wuhuangmontis *	China: Mt. Wuhuang, Pubei County, Guangxi	SYS a003486	MH605578
67	* Leptobrachella zhangyapingi *	Thailand: Chiang Mai	KJ-2013	JX069979
68	Leptobrachium cf. chapaense	Vietnam: Lao Cai	AMS R 171623	KR018126
69	* Pelobates syriacus *	/	MVZ234658	AY236807
70	* Pelobates varaldii *	/	/	AY236808
71	* Megophrys major *	Vietnam: Kon Tum	AMS R173870	KY476333

All specimens were fixed in 10 % buffered formalin and later transferred to 70 % ethanol for preservation, and deposited at the Museum of Biology, Sun Yat-sen University (**SYS**) and Chengdu Institute of Biology, the Chinese Academy of Sciences (**CIB**), China; tissue samples were preserved in 95% ethanol for molecular studies.

### DNA Extraction, PCR and sequencing

DNA was extracted from muscle tissue using a DNA extraction kit from Tiangen Biotech (Beijing) Co., Ltd. The mitochondrial gene 16S ribosomal RNA gene (16S rRNA) fragment from each sample was sequenced. Fragments were ampliﬁed using primer pairs L3975 (5’-CGCCTGTTTACCAAAAACAT-3’) and H4551 (5’-CCGGTCTGAACTCAGATCACGT-3’) (Simon et al. 1994). PCR ampliﬁcations were performed in a 20 μl reaction volume with the following cycling conditions: an initial denaturing step at 95 °C for five min; 35 cycles of denaturing at 95 °C for 40 s, annealing at 53 °C for 40 s and extending at 72 °C for one min; and a ﬁnal extending step of 72 °C for 10 min. PCR products were puriﬁed with spin columns. The purified products were sequenced with both forward and reverse primers using BigDye Terminator Cycle Sequencing Kit according to the guidelines of the manufacturer. The products were sequenced on an ABI Prism 3730 automated DNA sequencer in Shanghai Majorbio Bio-pharm Technology Co., Ltd.. All sequences have been deposited in GenBank (Table 1).

### Phylogenetic analyses

Sequences were first aligned in Clustal X 2.0 (Thompson et al. 1997), with default. The alignment was then checked and manually revised, if necessary. Trimmed with the gaps were partially deleted in MEGA 6.06 (Tamura et al. 2013), while within high variable regions, all gaps were removed.We ran Jmodeltest v2.1.2 (Darriba et al. 2012) with Akaike and Bayesian information criteria on the alignment, resulting the best-fitting nucleotide substitution models of GTR + I + G. Phylogenetic trees were constructed using maximum likelihood (ML) implemented in RaxmlGUI 1.3 (Silvestro and Michalak 2012), and Bayesian inference (BI) using MrBayes 3.2.4 (Ronquist et al. 2012). For ML analysis, the maximum likelihood tree inferred from 1000 replicates was used to represent the evolutionary history of the taxa analyzed. Branches corresponding to partitions reproduced in less than 60% of bootstrap replicates were collapsed. For BI analysis, two independent runs with four Markov Chain Monte Carlo simulations were performed for ten million iterations and sampled every 1000 iterations. The first 25% of samples were discarded as burn-in. Convergence of the Markov Chain Monte Carlo simulations was assessed with PSRF ≤0.01 and ESS (effective sample size) value > 200 using Tracer 1.4 (http://tree.bio.ed.ac.uk/software/tracer/). Pairwise distances were also calculated in MEGA 6.06 based on uncorrected *p*-distance (Tamura et al. 2013).

### Morphometrics

Measurements followed Fei et al. (2009) and Rowley et al. (2013), and were taken with a digital caliper to the nearest 0.1 mm. These measurements were as follows:

**SVL** snout-vent length (from tip of snout to vent);

**HDL** head length (from tip of snout to rear of jaws);

**HDW** head width (head width at commissure of jaws);

**SNT** snout length (from tip of snout to anterior corner of eye);

**EYE** eye diameter (diameter of exposed portion of eyeball);

**IOD** interorbital distance (minimum distance between upper eyelids);

**IND** internasal distance (distance between nares);

**TMP** tympanum diameter (horizontal diameter of tympanum);

**TEY** tympanum-eye distance (distance from anterior edge of tympanum to posterior corner of eye);

**TIB** tibia length (distance from knee to heel);

**ML** manus length (distance from tip of third digit to proximal edge of inner palmar tubercle);

**LAHL** length of lower arm and hand (distance from tip of the third finger to elbow);

**PL** pes length (distance from tip of fourth toe to proximal edge of the inner metatarsal tubercle);

**HLL** hindlimb length (distance from tip of fourth toe to vent).

Sex was determined by direct observation of calling in life, the presence of internal vocal sac openings, and the presence of eggs in abdomen seen via external inspection. Comparative morphological data of *Leptobrachella* species were obtained from examination of museum specimens (see Appendix 1) and from the references listed in Table 2. Due to the high likelihood of undiagnosed diversity within the genus (Rowley et al. 2016; Yang et al. 2016), where available, we rely on examination of topotypic material and/or original species descriptions.

**Table 2.** References for morphological characters for congeners of the genus *Leptobrachella*.

ID	*Leptobrachella* species	Literature obtained
**1**	*L.aereus* (Rowley, Stuart, Richards, Phimmachak & Sivongxay, 2010)	Rowley et al. 2010c
**2**	*L.alpinus* (Fei, Ye & Li, 1990)	Fei et al. 2009
**3**	*L.applebyi* (Rowley and Cao, 2009)	Rowley and Cao 2009
**4**	*L.arayai* (Matsui, 1997)	Matsui 1997
**5**	*L.ardens* (Rowley, Tran, Le, Dau, Peloso, Nguyen, Hoang, Nguyen & Ziegler, 2016)	Rowley et al. 2016
**6**	*L.baluensis* Smith, 1931	Dring 1983; Eto et al. 2016
**7**	*L.bidoupensis* (Rowley, Le, Tran & Hoang, 2011)	Rowley et al. 2011
**8**	*L.bondangensis* Eto, Matsui, Hamidy, Munir & Iskandar, 2018	Eto et al. 2018
**9**	*L.botsfordi* (Rowley, Dau & Nguyen, 2013)	Rowley et al. 2013
**10**	*L.bourreti* (Dubois, 1983)	Ohler et al. 2011
**11**	*L.brevicrus* Dring, 1983	Dring 1983; Eto et al. 2015
**12**	*L.crocea* (Rowley, Hoang, Le, Dau & Cao, 2010)	Rowley et al. 2010a
**13**	*L.dringi* (Dubois, 1987)	Inger et al. 1995; Matsui and Dehling 2012
**14**	*L.eos* (Ohler, Wollenberg, Grosjean, Hendrix, Vences, Ziegler & Dubois, 2011)	Ohler et al. 2011
**15**	*L.firthi* (Rowley, Hoang, Dau, Le & Cao, 2012)	Rowley et al. 2012
**16**	*L.fritinniens* (Dehling and Matsui, 2013)	Dehling and Matsui 2013
**17**	*L.fusca* Eto, Matsui, Hamidy, Munir & Iskandar, 2018	Eto et al. 2018
**18**	*L.fuliginosa* (Matsui, 2006)	Matsui 2006
**19**	*L.gracilis* (Günther, 1872)	Günther 1872; Dehling 2012b
**20**	*L.hamidi* (Matsui, 1997)	Matsui 1997
**21**	*L.heteropus* (Boulenger, 1900)	Boulenger 1900
**22**	*L.isos* (Rowley, Stuart, Neang, Hoang, Dau, Nguyen & Emmett, 2015)	Rowley et al. 2015a
**23**	*L.itiokai* Eto, Matsui & Nishikawa, 2016	Eto et al. 2016
**24**	*L.juliandringi* Eto, Matsui & Nishikawa, 2015	Eto et al. 2015
**25**	*L.kajangensis* (Grismer, Grismer & Youmans, 2004)	Grismer et al. 2004
**26**	*L.kalonensis* (Rowley, Tran, Le, Dau, Peloso, Nguyen, Hoang, Nguyen & Ziegler, 2016)	Rowley et al. 2016
**27**	*L.kecil* (Matsui, Belabut, Ahmad & Yong, 2009)	Matsui et al. 2009
**28**	*L.khasiorum* (Das, Tron, Rangad & Hooroo, 2010)	Das et al. 2010
**29**	*L.lateralis* (Anderson, 1871)	Anderson 1871; Humtsoe et al. 2008
**30**	*L.laui* (Sung, Yang & Wang, 2014)	Sung et al. 2014
**31**	*L.liui* (Fei and Ye, 1990)	Fei et al. 2009; Sung et al. 2014
**32**	*L.macrops* (Duong, Do, Ngo, Nguyen & Poyarkov, 2018)	Duong et al. 2018
**33**	*L.maculosa* (Rowley, Tran, Le, Dau, Peloso, Nguyen, Hoang, Nguyen & Ziegler, 2016)	Rowley et al. 2016
**34**	*L.mangshanensis* (Hou, Zhang, Hu, Li, Shi, Chen, Mo & Wang, 2018)	Hou et al. 2018
**35**	*L.maoershanensis* (Yuan, Sun, Chen, Rowley & Che, 2017)	Yuan et al. 2017
**36**	*L.marmorata* (Matsui, Zainudin and Nishikawa, 2014)	Matsui et al. 2014b
**37**	*L.maura* (Inger, Lakim, Biun and Yambun, 1997)	Inger et al. 1997
**38**	*L.melanoleuca* (Matsui, 2006)	Matsui 2006
**39**	*L.melica* (Rowley, Stuart, Neang & Emmett, 2010)	Rowley et al. 2010b
**40**	*L.minima* (Taylor, 1962)	Taylor 1962; Ohler et al. 2011
**41**	*L.mjobergi* Smith, 1925	Eto et al. 2015
**42**	*L.nahangensis* (Lathrop, Murphy, Orlov & Ho, 1998)	Lathrop et al. 1998
**43**	*L.natunae* (Günther, 1895)	Günther 1895
**44**	*L.nokrekensis* (Mathew and Sen, 2010)	Mathew and Sen 2010
**45**	*L.nyx* (Ohler, Wollenberg, Grosjean, Hendrix, Vences, Ziegler & Dubois, 2011)	Ohler et al. 2011
**46**	*L.oshanensis* (Liu, 1950)	Fei et al. 2009
**47**	*L.pallida* (Rowley, Tran, Le, Dau, Peloso, Nguyen, Hoang, Nguyen & Ziegler, 2016)	Rowley et al. 2016
**48**	*L.palmata* Inger and Stuebing, 1992	Inger and Stuebing 1992
**49**	*L.parva* Dring, 1983	Dring 1983
**50**	*L.pelodytoides* (Boulenger, 1893)	Boulenger 1893; Ohler et al. 2011
**51**	*L.petrops* (Rowley, Dau, Hoang, Le, Cutajar & Nguyen, 2017)	Rowley et al. 2017
**52**	*L.pictua* (Malkmus, 1992)	Malkmus 1992
**53**	*L.platycephala* (Dehling, 2012)	Dehling 2012a
**54**	*L.pluvialis* (Ohler, Marquis, Swan & Grosjean, 2000)	Ohler et al. 2000, 2011
**55**	*L.puhoatensis* (Rowley, Dau & Cao, 2017)	Rowley et al. 2016
**56**	*L.purpura* (Yang, Zeng & Wang, 2018)	Yang et al. 2018
**57**	*L.pyrrhops* (Poyarkov, Rowley, Gogoleva, Vassilieva, Galoyan & Orlov, 2015)	Poyarkov et al. 2015
**58**	*L.rowleyae* (Nguyen, Poyarkov, Le, Vo, Ninh, Duong, Murphy & Sang, 2018)	Nguyen et al. 2018
**59**	*L.sabahmontana* (Matsui, Nishikawa & Yambun, 2014)	Matsui et al. 2014a
**60**	*L.serasanae* Dring, 1983	Dring 1983
**61**	*L.sola* (Matsui, 2006)	Matsui 2006
**62**	*L.sungi* (Lathrop, Murphy, Orlov & Ho, 1998)	Lathrop et al. 1998
**63**	*L.tadungensis* (Rowley, Tran, Le, Dau, Peloso, Nguyen, Hoang, Nguyen & Ziegler, 2016)	Rowley et al. 2016
**64**	*L.tamdil* (Sengupta, Sailo, Lalremsanga, Das & Das, 2010)	Sengupta et al. 2010
**65**	*L.tengchongensis* (Yang, Wang, Chen & Rao, 2016)	Yang et al. 2016
**66**	*L.tuberosa* (Inger, Orlov & Darevsky, 1999)	Inger et al.1999
**67**	*L.ventripunctata* (Fei, Ye & Li, 1990)	Fei et al. 2009
**68**	*L.wuhuangmontis* Wang, Yang and Wang, 2018	Wang et al. 2018
**69**	*L.yingjiangensis* (Yang, Zeng & Wang, 2018)	Yang et al. 2018
**70**	*L.yunkaiensis* Wang, Li, Lyu and Wang, 2018	Wang et al. 2018
**71**	*L.zhangyapingi* (Jiang, Yan, Suwannapoom, Chomdej & Che, 2013)	Jiang et al. 2013

### Acoustic analyses

We compared the advertisement calls from three localities. One was in Wujing Nature Reserve and two were in Zhaozishan Nature Reserve. Advertisement calls were recorded between 20:00–24:00 h on 2–6 July 2018, using a Sony PCM-D100 digital sound recorder held within 20 cm of the calling individuals. The ambient temperature of the type locality was obtained using a Volt TP-2200 Humidity & Temperature Logger. The sound files in wave format were sampled at 44.1 kHz with sampling depth 24 bits. Praat 6.0.27 (Boersma 2001) was used to obtain the oscillograms, sonograms and power spectrums (window length = 0.005s). Raven pro 1.5 software (Bioacoustics Research Program 2013) was used to quantify the acoustic properties (window size = 256 points, fast Fourier transform, Hanning window). The measurements taken were as follows:

**Call Duration**: the time between onset of the first pulse and offset of the last pulse in a call;

**IQR (Inter-quartile Range)**: Duration of the difference between the 1^st^ and 3^rd^ quartile times which divides the selection into four time intervals containing equal energy in the selection;

**Dominant Frequency**: the frequency at which max power occurs within the selection;

**IQR (Inter-Quartile Range)**: Bandwidth of the difference between the 1^st^ and 3^rd^ quartile frequencies which divides the selection into four frequency intervals containing equal energy in the selection;

**fNote Pulses**: the number of pulses for the first note in a call;

**sNote Pulses**: the number of pulses for the second note in a call;

**Note Rise Time**: the time between onset of the first pulse and pulse of max amplitude;

**Note Interval**: the interval between the first note and the second note in a call;

**fNote Duration**: the duration of the first note in a call;

**sNote Duration**: the duration of the second note in a call.

Mean and standard deviation (SD) were calculated. We used median and interquartile range instead of mean and SD when calculating the undivided properties, like fNote Pulses and sNote Pulses. To identify different groups on acoustic properties, a hierarchical clustering using Mahalanobis distance was conducted (Mahalanobis 1936). The dendrogram was constructed based on Ward’s method (Ward Jr 1963). All statistical analyses were conducted in R 3.3.2 (R Core Team 2016).

## Results

### Molecular results

**Figure 2.**
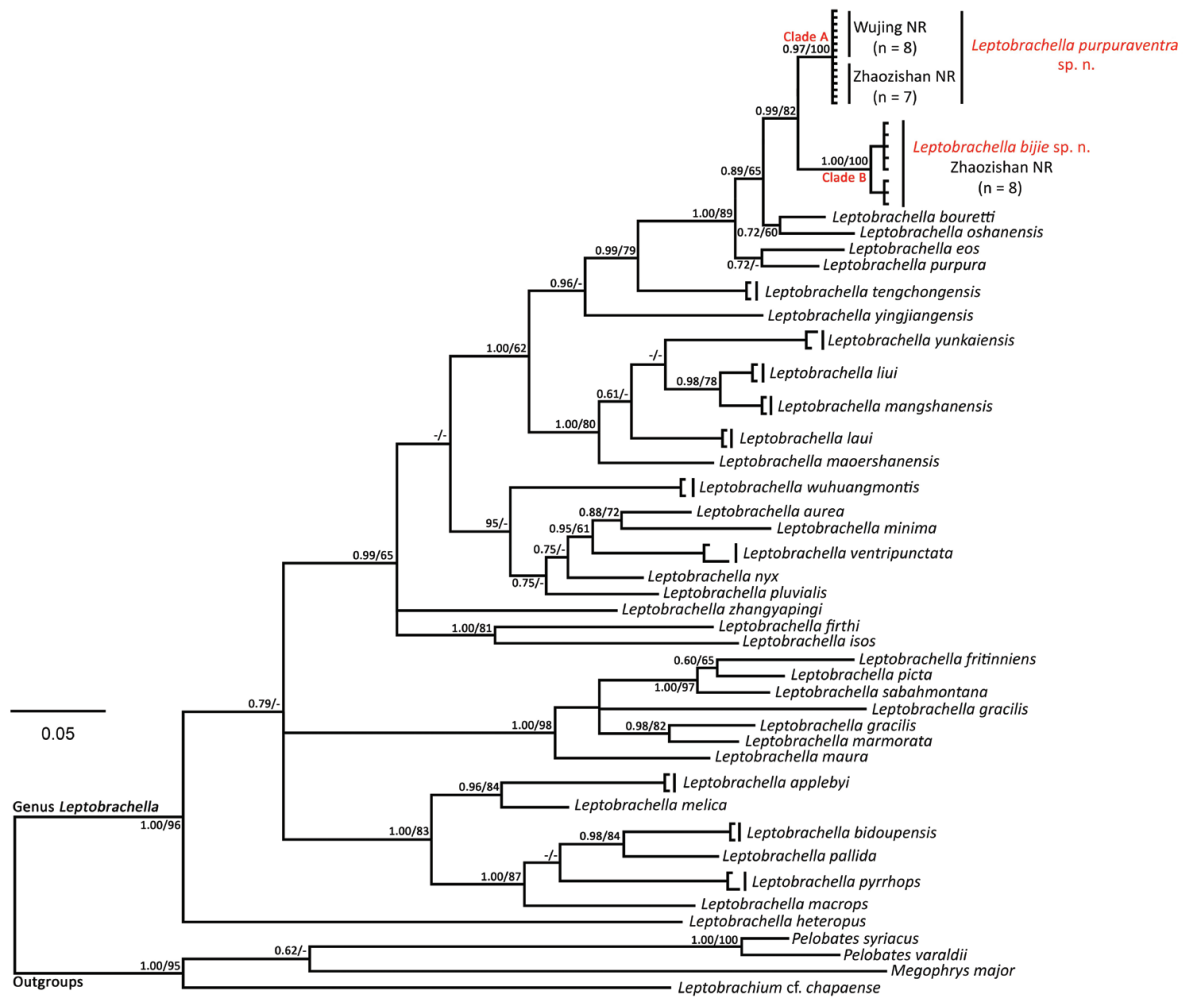
Bayesian inference tree of *Leptobrachella* species and out-groups derived from partial DNA sequences of the mitochondrial 16S r RNA gene. Numbers before slashes indicate Bayesian posterior probabilities (>0.6 retained) and numbers after slashes are bootstrap support for maximum likelihood (1000 replicates) analyses (>60 retained). The symbol “–” represents bootstrap value below 0.60/60%.

Bayesian inference (BI) and maximum likelihood (ML) phylogenetic trees were constructed based on DNA sequences of the mitochondrial 16S rRNA gene with a total length of 481-bp. The two analyses resulted in essentially identical topologies (Figure 2) which clustered the population of *Leptobrachella* from Jinjiazhai Village (JV) from Wujing Nature Reserve and those from Baimashan Forest Station (BFS) of Zhaozishan Nature Reserve together with very high node supporting values (0.97 in BI and 100% in ML) and represented a separately evolving lineage (Clade A). The population from Qingshan Village (QV) of Zhaozishan Nature Reserve (Clade B) was a sister taxon to Clade A with high node support values (0.99 in BI and 82% in ML). There was almost no genetic divergence between the two populations in Clade A even though the specimens were collected in two different sites with a straight-line distance at approximately 65 kilometers, and the smallest genetic divergence among individuals in Clade B was only 0.3%. The pairwise genetic divergence between Clade A and Clade B was 3.9–4.2%, and between Clade A and all other species of the genus *Leptobrachella* for which comparable sequences were included was 3.2% (between Clade A and *L.bourreti*), and between Clade B and all other species was 5.2–5.6% (between Clade B and *L.purpura*). However, these values were larger than or equal to observed pairwise genetic distances between recognized species (2.2% between *L.liui* and *L.mangshanensis*; 3.2% between *L.eos* and *L.purpura*) (Table 3).

**Table 3.** Uncorrected p-distances among *Leptobrachella* species based on 16S rRNA fragment (4 parts).

**Species & ID No.**	**(1)–(15)**	**(16)–(23)**	**(24)**	**(25)–(26)**	**(27)–(28)**	**(29)**	**(30)**	**(31)**	**(32)**
*Leptobrachellapurpuraventra* sp. nov. **(1)–(15)**	0								
*Leptobrachellabijie* sp. nov. **(16)–(23)**	3.9–4.2	0.0–0.3							
* Leptobrachella aerea * **(24)**	9.7	10.5–10.9	-						
* Leptobrachella applebyi * **(25)–(26)**	13.5	14.7–15.1	14.7	0					
* Leptobrachella bidoupensis * **(27)–(28)**	17	17.8–18.2	15.9	10.6	0				
* Leptobrachella bourreti * **(29)**	3.2	5.6–5.9	10.9	14.6	17.4	-			
* Leptobrachella eos * **(30)**	5.6	7.3–7.7	12	14.7	15.4	4.2	-		
* Leptobrachella firthi * **(31)**	14.6	14.2–14.6	13.1	16.6	18.7	13.5	13.8	-	
* Leptobrachella fritinniens * **(32)**	18.9	19.3–19.7	16.1	18.9	16.5	18.5	17.2	18.5	-
* Leptobrachella gracilis * **(33)**	22	23.3–23.8	20.7	18.1	21.8	21.5	22.8	24.1	13.1
* Leptobrachella hamidi * **(34)**	18.6	20.8–21.2	17.4	14.9	18.1	19.4	16.5	19	8.7
* Leptobrachella heteropus * **(35)**	21	23.2–23.7	18.1	17	18.9	21.5	21.4	22.6	19.6
* Leptobrachella isos * **(36)**	13.5	15.1–15.5	13.5	16.2	14.6	12	13.5	12.8	18.9
* Leptobrachella laui * **(37)–(38)**	11.3	10.9–11.2	11.3	16.7	17.4	10.9	10.2	14.7	18.9
* Leptobrachella liui * **(39)–(40)**	8.3	9.0–9.4	9.4	15.4	14.6	8.7	8	13.1	17.7
* Leptobrachella mangshanensis * **(41)–(42)**	9.7	10.5–10.8	10.5	16.2	15.7	10.1	9.4	15.1	19.3
* Leptobrachella marmorata * **(43)**	15.7	17.7–18.1	15.7	13	17.7	16.1	14.9	17.3	9.4
* Leptobrachella maura * **(44)**	16.5	17.0–17.4	17	17	17.7	18.2	17.4	18.5	10.8
* Leptobrachella macrops * **(45)**	15.4	17.9–18.3	14.2	12	9.8	15.8	15	17.8	16.9
* Leptobrachella maoershanensis * **(46)**	9.8	12.8–13.2	8.7	16.2	14.6	10.9	10.9	17.1	18.9
* Leptobrachella melica * **(47)**	11.9	14.3–14.7	11.7	6.3	10.3	13.8	15.1	16.6	16.9
* Leptobrachella minima * **(48)**	11.2	11.2–11.6	6.2	15	17	11.2	12	14.2	18.5
* Leptobrachella nyx * **(49)**	9	10.8–11.2	5.9	13.5	15	9.4	9.7	11.6	18.1
* Leptobrachella oshanensis * **(50)**	4.9	5.6–5.9	10.5	14.2	18.6	3.9	5.9	13.4	18.1
* Leptobrachella pallida * **(51)**	16.1	17.3–17.8	14.7	11.2	6.6	17.7	14.9	19	16.1
* Leptobrachella picta * **(52)**	18.5	19.7–20.2	17.3	16.1	17.7	18.1	17.2	17.3	5.6
* Leptobrachella pluvialis * **(53)**	9.7	11.9–12.3	5.2	14.6	15.4	10.1	11.2	14.2	18.4
* Leptobrachella purpura * **(54)**	4.3	5.2–5.6	10.1	13.9	14.6	3.9	3.2	13	16
* Leptobrachella ventripunctata * **(60)–(61)**	10.4–10.8	10.8–12.3	5.6	16.2–16.6	17.9–18.3	11.6–12.7	11.9–13.1	11.6–11.9	16.9–17.6
* Leptobrachella yingjiangensis * **(62)**	10.9	12.0–12.4	12.4	15.6	13.9	10.9	9.4	16.2	18.1
* Leptobrachella yunkaiensis * **(63)–(64)**	10.5–10.8	12.0–12.7	11.7–12	17.5	16.5–16.9	10.1–10.5	10.1–10.5	16.2	20.2–20.6
* Leptobrachella wuhuangmontis * **(65)–(66)**	13.1	14.3–14.7	8	16	15.4	11.2	12	13.9	19
* Leptobrachella zhangyapingi * **(67)**	11.7	12.0–12.4	10.3	15.5	16.2	11.3	10.1	13.1	19.8
**Part 2**									
**Species & ID No.**	**(33)**	**(34)**	**(35)**	**(36)**	**(37)–(38)**	**(39)–(40)**	**(41)–(42)**	**(43)**	**(44)**
* Leptobrachella gracilis * **(33)**	-								
* Leptobrachella hamidi * **(34)**	12.8	-							
* Leptobrachella heteropus * **(35)**	21.8	18.5	-						
* Leptobrachella isos * **(36)**	23.3	17.7	22.3	-					
* Leptobrachella laui * **(37)–(38)**	22.4	18.6	22.8	15.5	0				
* Leptobrachella liui * **(39)–(40)**	24.9	19.5	21.5	13.2	0.6	0			
* Leptobrachella mangshanensis * **(41)–(42)**	24.7	21.3	22.7	14.3	5.6	2.2	0		
* Leptobrachella marmorata * **(43)**	12.4	5.3	18.4	17.7	17.3	16.1	17.7	-	
* Leptobrachella maura * **(44)**	12	10.2	19.5	16.5	19.5	174	19.5	9.4	-
* Leptobrachella macrops * **(45)**	20.7	16.5	21.4	15.4	16.6	14.6	14.9	14.9	17
* Leptobrachella maoershanensis * **(46)**	24.3	20.4	21.9	15.1	7.7	6.3	6.2	17.7	19.5
* Leptobrachella melica * **(47)**	14.9	16.6	17.7	16.7	17.6	16.7	17.9	13.4	15
* Leptobrachella minima * **(48)**	21.5	19.5	19.4	14.3	9.8	9.4	9.7	16.9	17.8
* Leptobrachella nyx * **(49)**	23.7	17.3	18.1	13.1	9.8	8.3	9.7	15.7	17.3
* Leptobrachella oshanensis * **(50)**	20.2	19.4	22.8	12.7	8.7	8.3	9	17.3	17.7
* Leptobrachella pallida * **(51)**	19.7	16.9	20.5	18.3	15.4	15.3	15.7	15.3	16.5
**Part 3**									
**Species & ID No.**	**(45)**	**(46)**	**(47)**	**(48)**	**(49)**	**(50)**	**(51)**	**(52)**	**(53)**
* Leptobrachella macrops * **(45)**	-								
* Leptobrachella maoershanensis * **(46)**	15	-							
* Leptobrachella melica * **(47)**	11.3	16.3	-						
* Leptobrachella minima * **(48)**	16.1	9.4	14.6	-					
* Leptobrachella nyx * **(49)**	16.1	8.1	12.8	8	-				
* Leptobrachella oshanensis * **(50)**	16.6	11.3	14.2	9.4	9.7	-			
* Leptobrachella pallida * **(51)**	9	15.4	12.1	15	15.8	16.1	-		
* Leptobrachella picta * **(52)**	16.5	19.3	16.1	18	18.1	18.4	16.9	-	
* Leptobrachella pluvialis * **(53)**	16.1	7.3	14.7	7.2	5.9	10.5	14.6	18.4	-
* Leptobrachella purpura * **(54)**	14.6	10.1	14.3	11.6	9.4	5.6	14.5	17.3	9.7
* Leptobrachella pyrrhops * **(55)–(56)**	8.3–8.7	14.9–15.3	12.4–12.8	16.1–16.5	15.7–16.1	16.5–16.9	7.6–8.0	16.9–17.3	15.7–16.1
* Leptobrachella sabahmontana * **(57)**	15.3	19.9	13.7	16.9	18.9	17.3	14.9	5.2	19.3
* Leptobrachella tengchongensis * **(58)–(59)**	15.8	10.9	13.9	9.4	9.7	8.7	15.8	16.4	10.8
* Leptobrachella ventripunctata * **(60)–(61)**	17.8–18.1	9.4–9.8	15.1–15.4	6.2–7.2	6.2–6.6	10.9–12.0	16.6–17.0	17.6–18.4	6.9–7.2
**Part 4**									
**Species & ID No.**	**(54)**	**(55)–(56)**	**(57)**	**(58)–(59)**	**(60)–(61)**	**(62)**	**(63)–(64)**	**(65)–(66)**	**(67)**
* Leptobrachella purpura * **(54)**	-								
* Leptobrachella pyrrhops * **(55)–(56)**	15.7–16.5	0.3							
* Leptobrachella sabahmontana * **(57)**	16.9	16.1–16.4	-						
* Leptobrachella tengchongensis * **(58)–(59)**	8.7	16.1–16.5	16.5	0					
* Leptobrachella ventripunctata * **(60)–(61)**	10.8–11.9	16.1–16.2	16.5–16.6	16.5–17.3	9.4–10.5	0.9			
* Leptobrachella yingjiangensis * **(62)**	9.4	13.9–14.3	18.6	9.1	12.7	13.1	-		
* Leptobrachella yunkaiensis * **(63)–(64)**	10.1–10.5	16.2–16.5	16.6–17.0	21.1–21.5	12.4–12.7	11.6–11.9	0.3		
* Leptobrachella wuhuangmontis * **(65)–(66)**	12.4	16.2	16.6	19	13.9	9.8	10.9–15.6	0	
* Leptobrachella zhangyapingi * **(67)**	9.4	18.2	18.7	19	9.5	10.9	11.3	12.4	-

### Acoustic results

**Figure 3.**
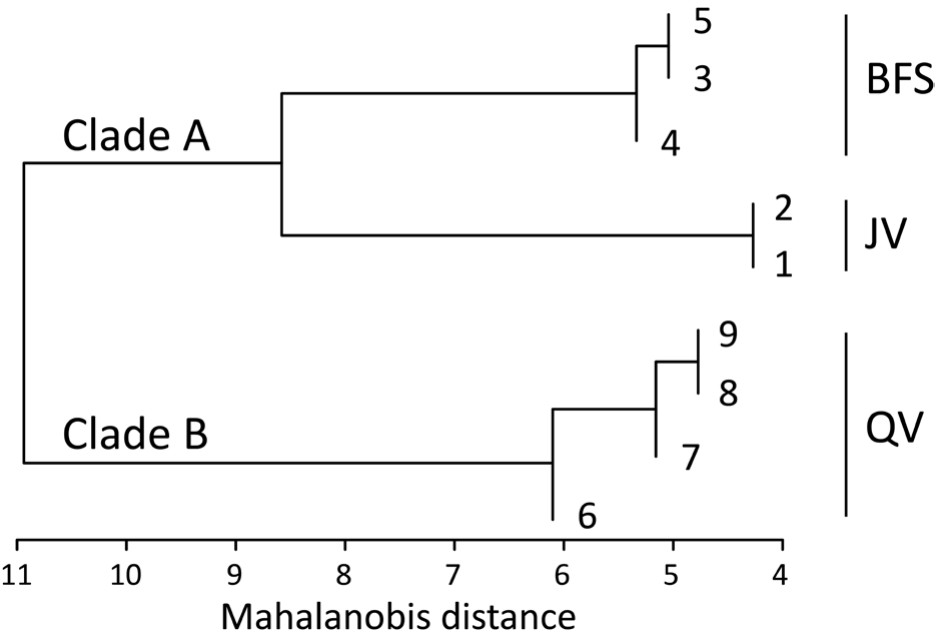
Hierarchical clustering of advertisement calls of *Leptobrachellapurpuraventra* sp. nov. from **BFS** Baimashan Forest Station in Zhaozishan Nature Reserve and **JV** Jinjiazhai Village in Wujing Nature Reserve, respectively; and *L.bijie* sp. nov. from **QV** Qingshan Village in Zhaozishan Nature Reserve.

Calling from nine male individuals were measured, respectively. They were recorded in Jinjiazhai Village (two males), Baimashan Forest Station (three males), and Qingshan Village (four males) at an ambient temperature approximately of 18.8 °C, 19.3 °C, and 18.6 °C, respectively. The result of hierarchical clustering analysis was consistent with the molecular result (Figure 3). Nine calling males were clustered into two clades based on acoustic properties of advertisement calls. All JV males and BFS males were clustered into Clade A, and all the QV males were clustered into Clade B. In Clade A, there were some differences in the advertisement calls between JV and BFS in Clade B. Measurements of the advertisement calls of the three localities are listed in Table 4.

**Table 4.** Measurements (mean ± standard deviation) of 11 acoustic properties for *Leptobrachella* species in this study.

**Locality**	**Individuals**	**Call Type**	**Call Duration (ms)**	**Call Interval (ms)**	**IQR Duration (ms)**	**Dominant Frequency (Hz)**	**IQR Bandwidth (Hz)**	fNote Pulses*	sNote Pulses*	**Note Rise Time (ms)**	**Note Interval (ms)**	**fNote Duration (ms)**	**sNote Duration (ms)**
*Leptobrachellapurpuraventra* sp. nov.													
**JV**	1	A (n=20)	111.6±3.0	119.1±8.0	30.8±27.3	4806.2±135.7	361.7±53.0	4±0	6±1	87.2±2.4	58.2±3.5	27.8±3.4	25.6±2.8
		B (n=14)	189.9±13.6	193.6±36.3	41.4±20.6	4835.7±196.6	319.9±148.9	4±1	20±2	69.3±9.6	42.6±11.2	24.7±5.6	122.5±11.4
	2	A (n=24)	110.7±5.0	128.5±13.7	57.8±23.1	4679.9±65.6	236.9±85.2	4±0	4±0	85.8±3.3	51.6±4.7	32.4±4.8	26.7±3.8
		B (n=18)	188±10.9	196.2±39.3	54.1±24.0	4679.9±66.0	306.2±73.7	3±0.75	19.5±1	57.3±30.1	43.1±9.1	25.3±5.9	119.7±7.4
**BFS**	3	A (n=20)	93.7±6.1	102.6±13.2	41.3±23.4	4823.4±0.0	180.9±38.5	3±0	4±1	70.5±3.1	45.0±4.2	24.5±2.8	24.2±6.0
		B (n=21)	192.2±13.0	174.5±51.1	50.4±20.1	4675.8±61.7	328.1±51.8	2±0	17±1	55.6±9.2	37.5±8.6	16.9±4.5	137.7±8.8
	4	A (n=20)	90.3±2.0	90.8±5.6	47.2±9.9	4823.4±0.0	344.5±0.0	3±0	4±0.25	69.4±2.0	39.4±2.2	28.2±1.1	22.8±2.4
		B (n=25)	191.8±11.9	174.5±51.1	60.6±16.6	4823.4±0.0	186.1±47.7	3±1	16±2	63.9±4.1	40.4±6.7	22.1±6.2	129.4±11.6
	5	A (n=20)	91.7±2.2	117.2±31.2	52.5±9.7	4720.1±86.6	198.1±63.1	3±0	4±0	67.4±3.4	40.3±4.7	25.2±3.7	26.2±3.4
		B (n=11)	144.8±31.5	217.8±64.9	27.2±7.1	4745.1±89.9	219.3±80.4	2±0.5	13±3.5	53.5±6.7	33.5±8.0	18.3±6.4	93.3±25.9
	Summary	A (n=104)	100.0±10.4	112.3±21.3	46.3±22.0	4767.1±97.3	263.4±92.8	3±1	4±1	76.4±9.1	47.1±8.1	27.8±4.4	25.2±4.1
		B (n=89)	185.0±21.7	182.7±47.9	49.7±21.4	4751.8±115.6	269.0±100.4	3±1	17±3	60.2±15.7	39.8±9.0	21.4±6.5	123.8±18.3
*Leptobrachellabijie* sp. nov.													
**QV**	6	A (n=26)	103.1±2.3	109.0±3.3	28.0±31.1	5068.6±86.8	344.5±0.0	2±0	3±0	87.8±2.1	70.1±7.8	16.4±7.2	16.6±0.8
		B (n=21)	221.0±14.0	235.6±45.1	38.1±20.5	5036.7±92.9	278.9±85.7	2±0	17±2	82.2±3.9	63.0±5.2	17.7±5.3	140.4±12.8
	7	A (n=25)	98.8±6.4	122.2±23.4	19.5±18.9	4823.4±0.0	172.3±0.0	3±1	4±1	76.4±3.7	55.9±4.9	19.3±4.6	23.5±6.5
		B (n=20)	206.8±10.1	245.2±79.4	56.6±20	4780.4±76.5	206.7±70.7	2±1	20±3	68.4±10.2	48.6±9.3	18.4±5.7	139.7±9.9
	8	A (n=22)	102.6±7.5	112.9±8.2	71.4±15.4	4909.6±88.2	172.3±0.0	3±0	4±1	85.3±5.2	61.3±5.9	22.5±1.6	18.9±3.2
		B (n=28)	253.0±19.0	225.6±61.1	71.5±34.9	4835.7±45.2	319.9±61.4	2±0.25	23±2.25	69.7±11.4	51.7±12.6	16.5±7.3	184.9±19.0
	9	A (n=33)	99.4±3.2	101.9±6.4	34.4±29.1	4823.4±0	302.8±74.9	2±1	2±1	83.0±2.9.0	60.1±6.6	21.7±6.6	17.7±1.9
		B (n=25)	264.0±12.5	166.5±44.5	64.6±23.5	4823.4±70.3	227.4±82.0	2±1	23±2	83.6±7.2	61.1±7.8	21.2±6.1	181.7±11.6
	Summary	A (n=106)	100.8±5.4	110.7±14.5	37.0±31.0	4901.4±116.8	255.2±86.4	3±1	3±2	83.1±5.4	61.8±8.2	20.0±6.0	19.0±4.5
		B (n=94)	239.0±27.0	216.3±65.4	59.0±28.7	4865.6±117.7	262.1±86.5	2±1	21.5±4	75.9±11.1	56.1±11.0	18.4±6.4	164.5±25.8

* Median instead of mean, inter-quartile range instead of SD. **JV**: Jinjiazhai Village; **BFS**: Baimashan Forest Station; **QV**: Qingshan Village.

**Figure 4.**
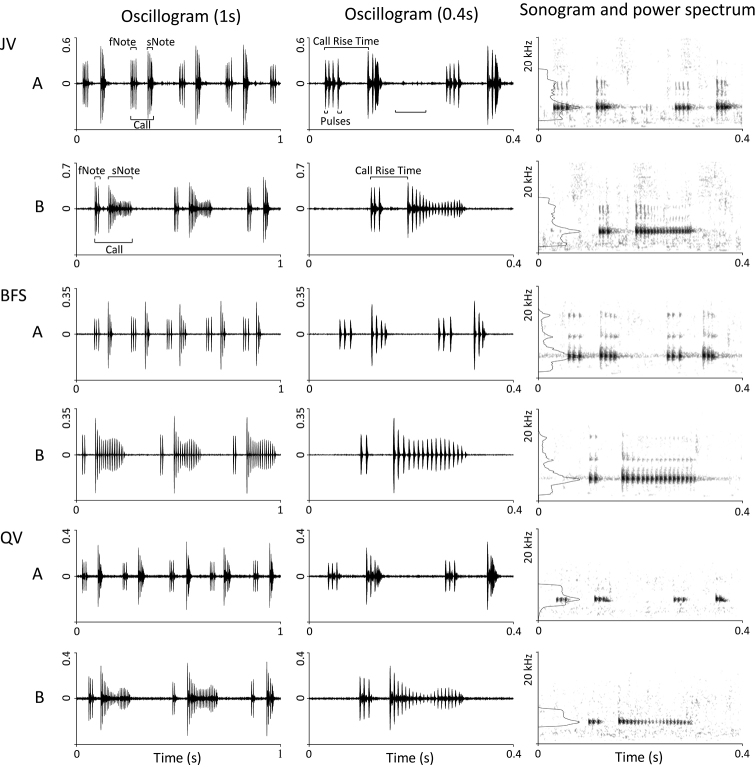
Different call types (**A** the first call type **B** the second call type.) of *Leptobrachellapurpuraventra* sp. nov. from **BFS** Baimashan Forest Station in Zhaozishan Nature Reserve and **JV** Jinjiazhai Village in Wujing Nature Reserve, respectively; and different call types of *L.bijie* sp. nov. from **QV** Qingshan Village in Zhaozishan Nature Reserve. (Window length: 0.005 s).

All advertisement calls contain two notes, each of which consists of repeated pulses (Figure 4). Clade A had more fNote pulses in second type of advertisement calls than those of Clade B (3 ± 1 vs. 2 ± 1), more sNote pulses in first type of advertisement calls (4 ± 1 vs. 3 ± 1), and less sNote pulses in the second type of advertisement calls (17 ± 3 vs. 21.5 ± 4). Accordingly, the sNote duration of Clade A was greater than those of Clade B (164.5 ± 25.8 vs. 123.8 ± 18.3 ms). Compared with individuals in Clade A, those from Clade B had little difference in the first type of advertisement calls, but had relatively short call duration (185.0 ± 21.7 vs. 239.0 ± 27.0 ms) and call interval (182.7 ± 47.9 vs. 216.3 ± 65.4 ms) in the second type. The dominant frequency of Clade A was higher than those of Clade B in both the first type of advertisement calls (4901.4 ± 116.8 vs. 4767.1 ± 97.3 Hz) and the second type (4865.6 ± 117.7 vs. 4751.8 ± 115.6 Hz).

Combining morphological, molecular genetics, and acoustic evidence, we herein describe these specimens as two new species.

### Taxonomy accounts

#### Leptobrachella bijie

Taxon classificationAnimaliaAnuraMegophryidae

J. Wang, Y.L. Li, Y. Li, H.H. Chen & Y.Y. Wang sp. nov.

http://zoobank.org/550E8562-0EC9-40C4-A6B3-FFAC35B25444

Figure 5

##### Holotype.

SYS a007316, adult male, collected by Jian Wang (JW hereafter) and Yulong Li (YLL hereafter) on 6 July 2018 from Qingshan Village (27°39'24"N, 105°23'14"E; 1670 m a.s.l.) in Zhaozishan Nature Reserve, Linkou Town, Qixingguan District, Bijie City, Guizhou Province, China.

##### Paratypes.

Seven adult males, SYS a007313/CIB 110002, SYS a007314–7315, 7317–7320, collected by Honghiu Chen (HHC hereafter), Yongyou Zhao (YYZ hereafter) and Jiahe Li (JHL), the same collection data as the holotype.

##### Diagnosis.

(1) small size (SVL 29.0–30.4 mm in eight adult males), (2) dorsal skin shagreened, some of the granules forming longitudinal short skin ridges, (3) iris bicolored, coppery orange on upper half and silver on lower half, (4) tympanum distinctly discernible, slightly concave, distinct black supratympanic line present, (5) internasal distance equal to interorbital distance, (6) supra-axillary, femoral, pectoral and ventrolateral glands distinctly visible, (7) absence of webbing and lateral fringes on fingers, toes with rudimentary webbing and narrow lateral fringes, (8) longitudinal ridges under toes not interrupted at the articulations, (9) relative finger lengths I = II = IV < III, relative toe length I < II < V = III < IV, (10) heels just meeting, tibia-tarsal articulation reaches the region between middle of eye to anterior corner of eye, (11) dorsal surface shagreened and granular, lacking enlarge tubercles or warts, some of the granules forming short longitudinal folds, (12) dorsum greyish-brown grounding, with small light orange granules, distinct darker brown markings scattered with irregular light orange pigmentations, (13) flanks with several dark blotches, longitudinally in two rows, (14) ventral surface white, with distinct nebulous greyish speckling on chest and ventrolateral flanks, (15) dorsal limbs including fingers and toes with dark bars, and (16) dense tiny conical spines present on surface of chest in males during breeding season.

**Figure 5.**
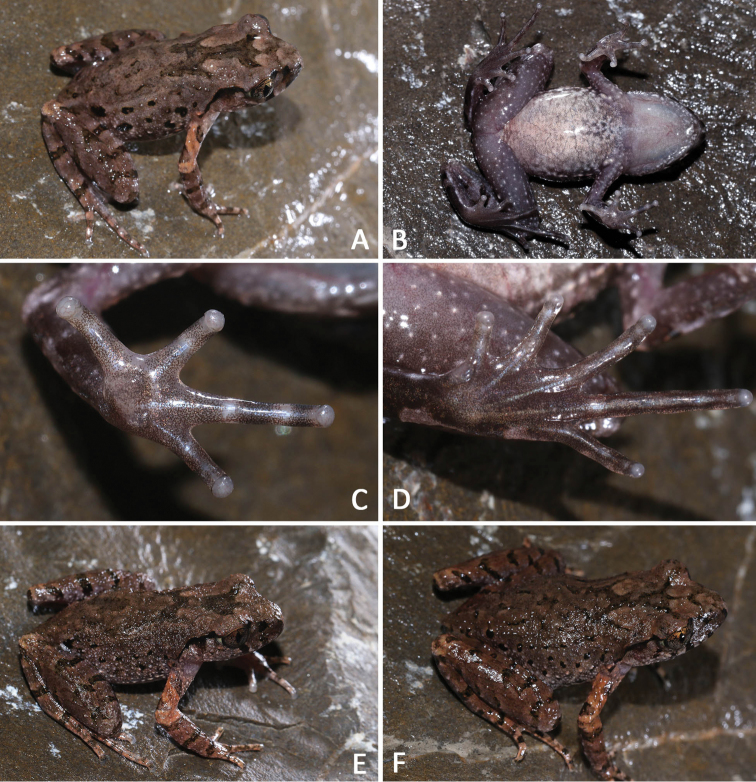
General aspect in life: **A–D**SYS a007316, the male holotype of *Leptobrachellabijie* sp. nov. **E** the male paratype SYS a007313 **F** the male paratype SYS a007317.

##### Comparisons.

Comparative morphological data of *Leptobrachellabijie* sp. nov. and 45 recognized *Leptobrachella* species occurring north of the Isthmus of Kra were listed in Table 5.

Compared with the 26 known congeners of the genus *Leptobrachella* occurring south of the Isthmus of Kra, by the presence of supra-axillary and ventrolateral glands, *L.bijie* sp. nov. can be easily distinguished from *L.arayai*, *L.dringi*, *L.fritinniens*, *L.gracilis*, *L.hamidi*, *L.heteropus*, *L.kajangensis*, *L.kecil*, *L.marmorata*, *L.melanoleuca*, *L.maura*, *L.picta*, *L.platycephala*, *L.sabahmontana*, and *L.sola*, all of which lacking supra-axillary and ventrolateral glands; and by the significantly larger body size, SVL 29.0–30.4 mm in males, *L.bijie* sp. nov. differs from the smaller *L.baluensis* (14.9–15.9 mm in males), *L.brevicrus* (17.1–17.8 mm in males), *L.bondangensis* (17.8 mm in male), *L.fusca* (16.3 mm in male), *L.itiokai* (15.2–16.7 mm in males), *L.juliandringi* (17.0–17.2 mm in males), *L.mjobergi* (15.7–19.0 mm in males), *L.natunae* (17.6 mm in one adult male), *L.parva* (15.0–16.9 mm in males), *L.palmata* (14.4–16.8 mm in males), *L.serasanae* (16.9 mm in female), and Dring’s (1983)*Leptobrachella* sp. 3 “*baluensis*” (15.0–16.0 mm in males).

**Table 5.** Selected diagnostic characters for species described herein and species in the genus *Leptobrachella* occurring north of the Isthmus of Kra (modified from Rowley et al. 2017; Yuan et al. 2017; Yang et al. 2018; Wang et al. 2018).

Species	Male SVL (mm)	Black spots on flanks	Toes webbing	Fringes on toes	Ventral coloration	Dorsal skin texture
*L.bijie* sp. nov.	29.0–30.4	Yes	Rudimentary	Narrow	White with distinct nebulous greyish speckling on chest and ventrolateral flanks	Shagreened and granular
*L.purpuraventra* sp. nov.	27.3–29.8	Yes	Rudimentary	Narrow	Grey purple with distinct nebulous greyish speckling on chest and ventrolateral flanks	Shagreened and granular
* L. aerea *	25.1–28.9	No	Rudimentary	Wide	Near immaculate creamy white, brown specking on margins	Finely tuberculate
* L. alpinus *	24.0–26.4	Yes	Rudimentary	Wide in males	Creamy-white with dark spots	Relatively smooth, some with small warts
* L. applebyi *	19.6–22.3	Yes	Rudimentary	No	Reddish brown with white speckling	Smooth
* L. ardens *	21.3–24.7	Yes	No	No	Reddish brown with white speckling	Smooth- finely shagreened
* L. bidoupensis *	18.5–25.4	Yes	Rudimentary	Weak	Reddish brown with white speckling	Smooth
* L. botsfordi *	29.1–32.6	No	Rudimentary	Narrow	Reddish brown with white speckling	Shagreened
* L. bourreti *	28.0–36.2	Yes	Rudimentary	Weak	Creamy white	Relatively smooth, some with small warts
* L. crocea *	22.2–27.3	No	Rudimentary	No	Bright orange	Highly tuberculate
* L. eos *	33.1–34.7	No	Rudimentary	Wide	Creamy white	Shagreened
* L. firthi *	26.4–29.2	No	Rudimentary	Wide in males	Creamy white	Shagreened with fine tubercles
* L. fuliginosa *	28.2–30.0	Yes	Rudimentary	Weak	White with brown dusting	Nearly smooth, few tubercles
* L. isos *	23.7–27.9	No	Rudimentary	Wide in males	Creamy white with white dusting on margins	Mostly smooth, females more tuberculate
* L. kalonensis *	25.8–30.6	Yes	No	No	Pale, speckled brown	Smooth
* L. khasiorum *	24.5–27.3	Yes	Rudimentary	Wide	Creamy white	Isolated, scattered tubercles
* L. lateralis *	26.9–28.3	Yes	Rudimentary	No	Creamy white	Roughly granular
* L. laui *	24.8–26.7	Yes	Rudimentary	Wide	Creamy white with dark brown dusting on margins	Round granular tubercles
* L. liui *	23.0-28.7	Yes	Rudimentary	Wide	Creamy white with dark brown spots on chest and margins	Round granular tubercles with glandular folds
* L. macrops *	28.0–29.3	Yes	Rudimentary	No	Greyish-violet with white speckling	Roughly granular with larger tubercles
* L. maculosa *	24.2–26.6	Yes	No	No	Brown, less white speckling	Mostly smooth
* L. maoershanensis *	25.2–30.4	Yes	Rudimentary	Narrow	Creamy white chest and belly with irregular black spots	Longitudinal folds
* L. mangshanensis *	22.22–27.76	Yes	Rudimentary	Weak	White speckles on throat and belly	Nearly smooth
* L. melica *	19.5–22.7	Yes	Rudimentary	No	Reddish brown with white speckling	Smooth
* L. minima *	25.7–31.4	Yes	Rudimentary	No	Creamy white	Smooth
* L. nahangensis *	40.8	Yes	Rudimentary	No	Creamy white with light specking on throat and chest	Smooth
* L. nokrekensis *	26.0–33.0	Yes	Rudimentary	unknown	Creamy white	Tubercles and longitudinal folds
* L. nyx *	26.7–32.6	Yes	Rudimentary	No	Creamy white with white with brown margins	Rounded tubercles
* L. oshanensis *	26.6–30.7	Yes	No	No	Whitish with no markings or only small, light grey spots	Smooth with few glandular ridges
* L. pallida *	24.5–27.7	No	No	No	Reddish brown with white speckling	Tuberculate
* L. pelodytoides *	27.5–32.3	Yes	Wide	Narrow	Whitish	Small, smooth warts
* L. petrops *	23.6–27.6	No	No	Narrow	Immaculate creamy white	Highly tuberculate
* L. pluvialis *	21.3–22.3	Yes	Rudimentary	No	Dirty white with dark brown marbling	Smooth, flattened tubercles on flanks
* L. puhoatensis *	24.2–28.1	Yes	Rudimentary	Narrow	Reddish brown with white dusting	Longitudinal skin ridges
* L. purpura *	25.0–27.5	Yes	Rudimentary	Wide	Dull white with indistinct grey dusting	Shagreen with small tubercles
* L. pyrrhops *	30.8–34.3	Yes	Rudimentary	No	Reddish brown with white speckling	Slightly shagreened
* L. rowleyae *	23.4–25.4	Yes	No	No	Pinkish milk-white to light brown chest and belly with numerous white speckles	Smooth with numerous tiny tubercles
* L. sungi *	48.3–52.7	No or small	Wide	Weak	White	Granular
* L. tadungensis *	23.3–28.2	Yes	No	No	Reddish brown with white speckling	Smooth
* L. tamdil *	32.3	Yes	Wide	Wide	White	Weakly tuberculate
* L. tengchongensis *	23.9–26.0	Yes	Rudimentary	Narrow	White with dark brown blotches	Shagreened with small tubercles
* L. tuberosa *	24.4–29.5	No	Rudimentary	No	White with small grey spots/streaks	Highly tuberculate
* L. ventripunctata *	25.5–28.0	Yes	Rudimentary	No	Chest and belly with dark brown spots	Longitudinal skin ridges
* L. wuhuangmontis *	25.6–30.0	Yes	Rudimentary	Narrow	Greyish white mixed by tiny white and black dots	Rough, scattered with dense conical tubercles
* L. yingjiangensis *	25.7–27.6	Yes	Rudimentary	Wide	Creamy white with dark brown flecks on chest and margins	Shagreened with small tubercles
* L. yunkaiensis *	25.9–29.3	Yes	Rudimentary	Wide	Belly pink with distinct or indistinct speckling	Shagreened with short skin ridges and raised warts
* L. zhangyapingi *	45.8–52.5	No	Rudimentary	Wide	Creamy-white with white with brown margins	Mostly smooth with distinct tubercles

For the remaining 45 members of the genus *Leptobrachella*, having SVL of 29.0–30.4 mm in males, *L.bijie* sp. nov. differs from the larger *L.eos* (33.1–34.7 mm in males), *L.nahangensis* (40.8 mm in male), *L.sungi* (48.3–52.7 mm in males), *L.tamdil* (32.3 mm in male), and *L.zhangyapingi* (45.8–52.5 mm in males); and from the smaller *L.alpinus* (24.0–26.4 mm in males), *L.applebyi* (19.6–22.3 mm in males), *L.ardens* (21.3–24.7 mm in males), *L.bidoupensis* (18.5–25.4 mm in males), *L.crocea* (22.2–27.3 mm in males), *L.isos* (23.7–27.9 mm in males), *L.khasiorum* (24.5–27.3 mm in males), *L.lateralis* (26.9–28.3 mm in males), *L.laui* (24.8–26.7 mm in males), *L.maculosa* (24.2–26.6 mm in males), *L.melica* (19.5–22.7 mm in males), *L.pallida* (24.5–27.7 mm in males), *L.petrops* (23.6–27.6 mm in males), *L.pluvialis* (21.3–22.3 mm in males), *L.puhoatensis* (24.2–28.1 mm in males), *L.purpura* (25.0–27.5 mm in males), *L.rowleyae* (23.4–25.4 mm in males), *L.tadungensis* (23.3–28.2 mm in males), *L.tengchongensis* (23.9–26.0 mm in males), *L.ventripunctata* (25.5–28.0 mm in males), and *L.yingjiangensis* (25.7–27.6 mm in males).

In having black spots on flanks, the new species differs from *L.aerea*, *L.botsfordi*, *L.firthi*, and *L.tuberosa*, all of which lacking distinct black spots on the flanks; by having rudimentary webbing on toes, the new species differs from *L.kalonensis* and *L.oshanensis*, both of which lacking webbing on toes, and from *L.pelodytoides*, which bears wide webbing on toes; by having narrow lateral fringes on toes, the new species differs from *L.aerea*, *L.firthi*, *L.liui*, and *L.yunkaiensis*, all of which having wide lateral fringes on toes, from *L.bourreti* and *L.fuliginosa*, both of which having weak lateral fringes on toes, and from *L.kalonensis*, *L.macrops*, *L.minima*, *L.nyx*, *L.oshanensis*, *L.pyrrhops*, and *L.tuberosa*, all of which lacking lateral fringes on toes; by having dorsal surface shagreened and granular, lacking enlarge tubercles or warts, the new species differs from *L.bourreti* (dorsum smooth with small warts), *L.fuliginosa* (dorsum smooth with fine tubercles), *L.liui* (dorsum with round tubercles), *L.macrops* (dorsum roughly granular with large tubercles), *L.maoershanensis* (dorsum shagreened with tubercles), *L.minima* (dorsum smooth), *L.nyx* (dorsum with round tubercles), *L.pelodytoides* (dorsum with small, smooth warts), *L.tuberosa* (dorsum hingly tuberculate), *L.yunkaiensis* (dorsum with raised warts), and *L.wuhuangmontis* (dorsum rough with conical tubercles); by having ventral surface white with distinct nebulous greyish speckling on chest and flanks, the new species differs from *L.botsfordi* and *L.pyrrhops*, (ventral reddish brown with white speckling), *L.maoershanensis* (belly with irregular black spots); by having tiny spines on surface of chest in males during breeding season, the new species differs from all male specimens collected in breeding season of *L.liui*, *L.oshanensis*, *L.yunkaiensis*, and *L.wuhuangmontis*, all of which are lacking such spines.

##### Description of holotype.

Adult male. Body size small, SVL in 29.3 mm. Head length slightly larger than head width, HDL/HDW 1.03; snout slightly protruding, projecting slightly beyond margin of the lower jaw; nostril closer to snout than eye; canthus rostralis gently rounded; loreal region slightly concave; interorbital space flat, internarial distance equal to interorbital distance, IND/IOD 1.00; pineal ocellus absent; vertical pupil; snout length larger than eye diameter, SNT/EYE 1.11; tympanum distinct, rounded, and slightly concave, diameter smaller than that of the eye and larger than tympanum-eye distance, TMP/EYE 0.53 and TEY/TMP 0.47; upper margin of tympanum incontact with supratympanic ridge; distinct black supratympanic line present; vomerine teeth absent; vocal sac openings slit-like, paired, located posterolaterally on floor of mouth in close proximity to the margins of the mandible; tongue deeply notched behind; supratympanic ridge distinct, extending from posterior corner of eye to supra-axillary gland.

Tips of fingers rounded, slightly swollen; relative finger lengths I = II = IV < III; nuptial pad absent; subarticular tubercles absent; a large, rounded inner palmar tubercle distinctly separated from small, round outer palmar tubercle; absence of webbing and lateral fringes on fingers. Tips of toes like fingers; relative toe length I < II < V = III < IV; subarticular tubercles absent; distinct dermal ridges present under the 3^rd^ to 5^th^ toes, not interrupted; large, oval inner metatarsal tubercle present, outer metatarsal tubercle absent; toes webbing rudimentary; narrow lateral fringes present on all toes. Tibia 47% of snout-vent length; tibiotarsal articulation reaches to middle of eye; heels just meeting each other when thighs are appressed at right angles with respect to body.

Dorsal surface shagreened and granular, lacking enlarge tubercles or warts, some of the granules forming short longitudinal folds; ventral skin smooth; dense tiny conical spines present on surface of chest; pectoral gland and femoral gland oval; pectoral glands greater than tips of fingers and femoral glands; femoral gland situated on posteroventral surface of thigh, closer to knee than to vent; supra-axillary gland raised. Ventrolateral gland distinctly visible, forming an incomplete line.

##### Measurements of holotype (in mm).

SVL 29.2, HDL 10.0, HDW 9.7, SNT 4.0, EYE 3.6, IOD 3.0, IND 3.0, TMP 1.9, TEY 0.9, TIB 13. 8, ML 7.8, PL 13.2, LAHL 14.1, HLL 43.3.

##### Coloration of holotype in life.

Dorsum greyish-brown grounding, with small reddish granules, distinct darker brown markings and rounded spots and scattered with irregular light orange pigmentation. A dark brown inverted triangular pattern between anterior corner of eyes, in connected to the dark brown W-shaped marking on interorbital region, and the W-shaped marking in connected to the other W-shaped marking between axillae. Tympanum brown. Small light orange granules present on dorsum of body and limb; a dark brown vertical bar under the eye; transverse dark brown bars on dorsal surface of limbs; distinct dark brown blotches on flanks from groin to axilla, longitudinally in two rows; elbow and upper arms with dark bars and distinct coppery orange coloration; fingers and toes with distinct dark bars.

Ventral surface of throat, chest, and belly white, presence of distinct nebulous greyish speckling on chest and ventrolateral flanks; ventral surface of limbs grey purple. Supra-axillary gland coppery orange; femoral, pectoral and ventrolateral glands greyish white. Iris bicolored, coppery orange on upper half and silver on lower half.

##### Coloration of holotype in preservative.

Dorsum of body and limbs dark brown; transverse bars on limbs become more distinct; dark brown patterns, markings and spots on back become indistinct, orange pigmentations become greyish white. Ventral surface of body and limbs greyish white, nebulous speckling on chest and flanks balck brown. Supra-axillary, femoral, pectoral and ventrolateral glands greyish white.

##### Variations.

Measurements and body proportions were listed in Table 6. All paratypes match the overall characters of the holotype except that: coloration of tympanum brown in the holotype SYS a007316 (vs. black in paratypes SYS a007313/CIB 110002 (Figure 5E), SYS a007315, 7317 (Figure 5F)); heels just meeting, tibia-tarsal articulation reaching the middle of eye in the holotype (vs. heels slightly overlapping in paratypes SYS a007315, 7317, 7319–7320; tibia-tarsal articulation reaching the anterior corner of eye in paratypes SYS a007315, 7317, 7319); W-shaped marking on interorbital region in connected to the other W-shaped marking between axillae in the holotype (vs. such markings not in connected with each other in paratypes SYS a007313/CIB 110002, SYS a007320); a dark brown inverted triangular pattern between anterior corner of eyes in the holotype (vs. a V-shaped pattern between anterior corner of eyes instead in paratype SYS a007317, 7320); relatively larger black spots on flanks (vs. black spots distinctly small in paratypes SYS a007313/CIB 110002, SYS a007317).

**Table 6.** Measurements (minimum–maximum (mean ± SD); in mm), and body proportions of *Leptobrachellabijie* sp. nov. from Qingshan Village of Zhaozishan Nature Reserve.

**SEX**	**Males (n = 8)**		
**SVL**	29.0–30.4 (29.7 ± 0.6)	**HLL**	43.0–45.5 (43.7 ± 0.8)
**HDL**	10.0–10.6 (10.2 ± 0.2)	**HDL/HDW**	1.02–1.05 (1.04 ± 0.01)
**HDW**	9.5–10.2 (9.8 ± 0.3)	**HDL/SVL**	0.33–0.35 (0.34 ± 0.01)
**SNT**	4.0–4.7 (4.3 ± 0.3)	**SNT/HDL**	0.40–0.44 (0.42 ± 0.02)
**EYE**	3.6–4.1 (3.8 ± 0.2)	**SNT/EYE**	1.11–1.15 (1.13 ± 0.02)
**IOD**	2.8–3.4 (3.1 ± 0.2)	**EYE/TMP**	1.85–1.95 (1.89 ± 0.04)
**IND**	2.8–3.4 (3.1 ± 0.2)	**IND/IOD**	1
**TMP**	1.9–2.2 (2.0 ± 0.1)	**TMP/EYE**	0.51–0.54 (0.53 ± 0.01)
**TEY**	0.9–1.1 (1.0 ± 0.1)	**TEY/TMP**	0.45–0.53 (0.48 ± 0.02)
**TIB**	13.5–14.4 (13.8 ± 0.3)	**TIB/SVL**	0.45–0.47 (0.47 ± 0.01)
**ML**	7.4–8.3 (7.8 ± 0.3)	**LAHL/SVL**	0.47–0.49 (0.48 ± 0.01)
**PL**	13.0–13.8 (13.3 ± 0.2)	**HLL/SVL**	1.45–1.50 (1.47 ± 0.02)
**LAHL**	14.0–14.8 (14.3 ± 0.3)	**TIB/HLL**	0.31–0.32 (0.31 ± 0.01)

##### Etymology.

The specific epithet *bijie* is in reference to the type locality, Qingshan Village in Bijie City of Guizohu Province, China. For the common name, we suggest “Bijie Leaf Litter Toad”, and for the Chinese name “Bi Jie Zhang Tu Chan (毕节掌突蟾)”.

##### Distribution and habits.

**Figure 6.**
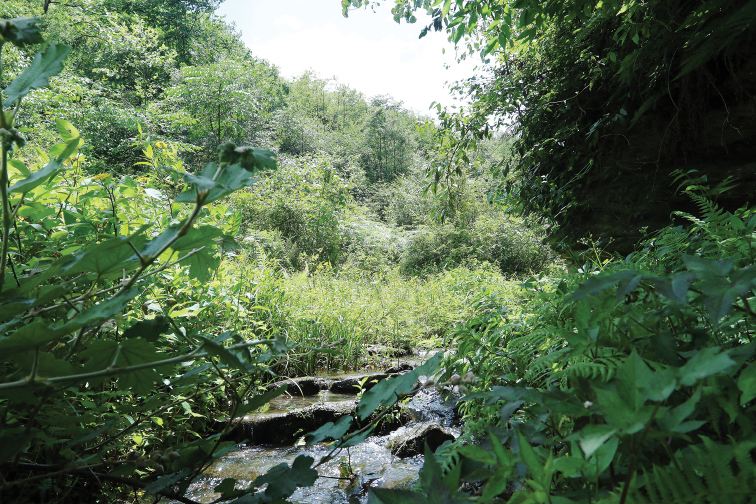
The habitat of *Leptobrachellabijie* sp. nov. in Qingshan Village of Zhaozishan Nature Reserve in Guizhou Province.

Currently, *Leptobrachellabijie* sp. nov. is known only from its type locality Qingshan Village in Zhaozishan Nature Reserve, Linkou County, Qixingguan District, Bijie City, Guizhou Province, China (Figure 1). The new species was found along a clear-water rocky stream (ca. 2 m in width and ca. 20–30 cm in depth; 1670–1750 m a.s.l.) in karst landforms. The stream was surrounded by broad-leaved forest at an altitude below 1700 m, and by coniferous forest at an altitude above 1700 m (Figure 6, 1700 m a.s.l.). On 6 July 2018 at 22:00–23:30 P.M., a large number of males were found calling on leaves of plants (Figure 10A), and some were found calling perching on the rocks or under rocks by the side of the stream.

#### Leptobrachella purpuraventra

Taxon classificationAnimaliaAnuraMegophryidae

J. Wang, Y.L. Li, Y. Li, H.H. Chen & Y.Y. Wang sp. nov.

http://zoobank.org/0B2C4A25-981B-4AE9-900D-60CAB4E7A560

Figure 7

##### Holotype.

SYS a007284, adult male, collected by JW on 2 July 2018 from Jinjiazhai Village (27°7'5.92"N, 105°19'28.47"E; 1890 m a.s.l.) in Wujing Nature Reserve, Chahe Town, Qixingguan District, Bijie City, Guizhou Province, China.

##### Paratypes.

A single adult female, SYS a007278 and seven adult males, SYS a007277/CIB 110003, 7279–7284, collected by JW, YLL, YYZ, HHC, JHL and Yingyong Wang (YYW hereafter), the same collection data as the holotype; besides, another three adult females, SYS a007304–7306, and four adult males, SYS a007300–7303, collected by JW, YLL, YYZ, HHC, JHL and YYW on 4 July 2018 from Baimashan Forest Station (27°41'25"N, 105°27'16"E; 1600 m a.s.l.) of Zhaozishan Nature Reserve, Shengji Town, Qixingguan District, Bijie City, Guizhou Province, China.

##### Diagnosis.

(1) small size (SVL 27.3–29.8 mm in males, 33.0–35.3 mm in females), (2) dorsal skin shagreened, some of the granules forming longitudinal short skin ridges, (3) iris bicolored, coppery orange on upper half and silver on lower half, (4) tympanum distinctly discernible, slightly concave, distinct black supratympanic line present, (5) internasal distance smaller than interorbital distance, IND/IOD ratio 1.03–1.10, (6) supra-axillary, femoral, pectoral and ventrolateral glands distinctly visible, (7) absence of webbing and lateral fringes on fingers, toes with rudimentary webbing and narrow lateral fringes, (8) longitudinal ridges under toes not interrupted at the articulations, (9) heels just meeting or slightly overlapping, tibia-tarsal articulation reaching to the middle of eye, (10) relative finger lengths I = II = IV < III, relative toe length I < II < V < III < IV, (11) dorsal surface shagreened and granular, lacking enlarge tubercles or warts, some of the granules forming short longitudinal folds, (12) dorsum purple brown to dark purple brown or grey purple grounding, with small light orange granules, distinct darker brown markings scattered with irregular light orange pigmentations, (13) flanks with several dark blotches, longitudinally in two rows, (14) ventral surface grey purple, with distinct or indistinct nebulous greyish speckling on chest and ventrolateral flanks, without black spots (seldom present), (15) dorsal limbs including fingers and toes with dark bars, those on forearms indistinct, and (16) dense tiny conical spines present on surface of chest extending to anterior region of abdomen in males, and absent in females during breeding season.

**Figure 7.**
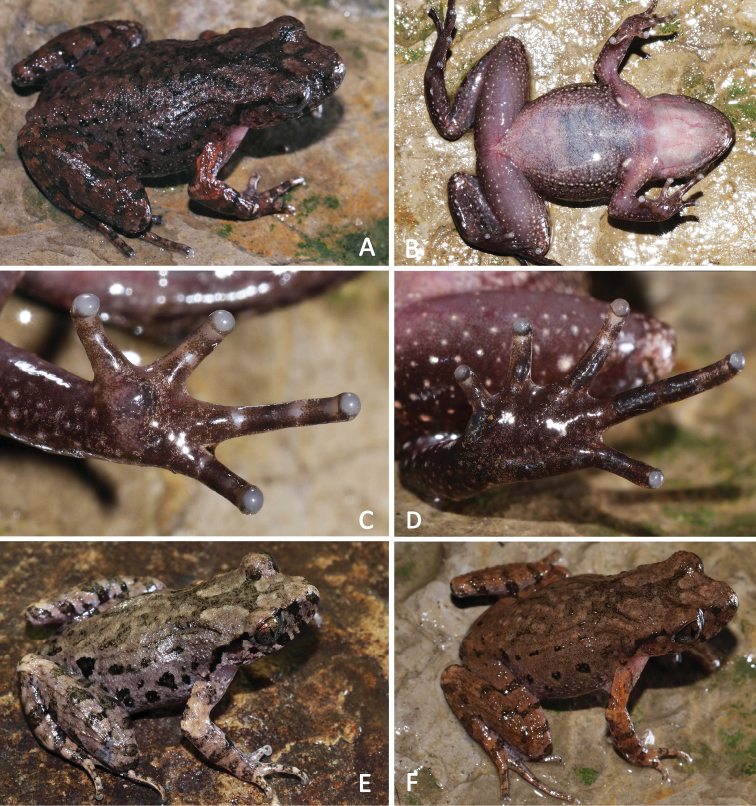
General aspect in life: **A–D**SYS a007284, the male holotype of *Leptobrachellapurpuraventra* sp. nov. **E** the male paratype SYS a007300 **F** the male paratype SYS a007283.

##### Comparisons.

Comparative morphological data of *Leptobrachellapurpuraventra* sp. nov., *L.bijie* sp. nov., and 45 recognized *Leptobrachella* species occurring north of the Isthmus of Kra were listed in Table 5.

In the phylogenetic trees (Figure 2), *Leptobrachellapurpuraventra* sp. nov. is a sister taxon to *L.bijie* sp. nov. with a high support value (99% in BI, 0.82 in ML), and it can be distinguished from the later by a genetic divergence (*p*=3.9–4.2%). Morphologically, it differs from the later by the coloration of dorsum and ventral, dorsum purple brown to dark purple brown or grey purple grounding, ventral grey purple grounding (vs. dorsum greyish-brown grounding, ventral white grounding); dark bars on dorsal limbs indistinct (vs. distinctly visible); dark bars on dorsal surface of tibia and tarsus much broader, especially those on dorsal skin of tarsus (vs. relatively narrow dark bars on dorsal surface of tibia and tarsus); internasal distance smaller than interorbital distance, IND/IOD ratio 1.03–1.10 (vs. internasal distance equal to interorbital distance, IND/IOD ratio 1.00); larger TEY value, TEY/TMP ratio 0.60–0.76 (vs. TEY/TMP ratio 0.45–0.53); dense tiny conical spines present on surface of chest extending to anterior region of abdomen (vs. such spines less developed, present on surface of chest, not extending to anterior region of abdomen); lateral fringes on toes narrow but more developed and distinct (vs. less developed); length of toe V < III (length of toe V = III).

Compared with the 26 known congeners of the genus *Leptobrachella* occurring south of the Isthmus of Kra, by the presence of supra-axillary and ventrolateral glands, *L.purpuraventra* sp. nov. can be easily distinguished from *L.arayai*, *L.dringi*, *L.fritinniens*, *L.gracilis*, *L.hamidi*, *L.heteropus*, *L.kajangensis*, *L.kecil*, *L.marmorata*, *L.melanoleuca*, *L.maura*, *L.picta*, *L.platycephala*, *L.sabahmontana*, and *L.sola*, all of which lacking supra-axillary and ventrolateral glands; and by the significantly larger body size, SVL 27.3–29.8 mm in males, 33.0–35.3 mm in females, *L.purpuraventra* sp. nov. differs from the smaller *L.baluensis* (14.9–15.9 mm in males), *L.bondangensis* (17.8 mm in male), *L.brevicrus* (17.1–17.8 mm in males), *L.fusca* (16.3 mm in male), *L.itiokai* (15.2–16.7 mm in males), *L.juliandringi* (17.0–17.2 mm in males and 18.9–19.1 mm in females), *L.mjobergi* (15.7–19.0 mm in males), *L.natunae* (17.6 mm in male), *L.parva* (15.0–16.9 mm in males and 17.8 mm in female), *L.palmata* (14.4–16.8 mm in males), *L.serasanae* (16.9 mm in female), and Dring’s (1983)*Leptobrachella* sp. 3 “*baluensis*” (15.0–16.0 mm in males).

For the remaining 45 members of the genus *Leptobrachella*, in having SVL 27.3–29.8 mm in males and 33.0–35.3 mm in females, *L.purpuraventra* sp. nov. differs from the larger *L.bourreti* (42.0–45.0 mm in females), *L.eos* (33.1–34.7 mm in males and 40.7 in female), *L.lateralis* (36.6 mm in females), *L.nahangensis* (40.8 mm in male), *L.nyx* (37.0–41.0 mm in females), *L.sungi* (48.3–52.7 mm in males and 56.7–58.9 mm in females), *L.tamdil* (32.3 mm in male), and *L.zhangyapingi* (45.8–52.5 mm in males); and from the smaller *L.alpinus* (24.0–26.4 mm in males), *L.applebyi* (19.6–22.3 mm in males and 21.7–26.4 mm in females), *L.ardens* (21.3–24.7 mm in males, 24.5 mm in female), *L.bidoupensis* (18.5–25.4 mm in males), *L.kalonensis* (28.9–30.6 mm in females), *L.maculosa* (27.0 mm in female), *L.maoershanensis* (29.1 mm in female), *L.mangshanensis* (30.2 mm in female), *L.melica* (19.5–22.7 mm in males), *L.pluvialis* (21.3–22.3 mm in males), *L.rowleyae* (23.4–25.4 mm in males), *L.tadungensis* (32.1 mm in female), and *L.tengchongensis* (23.9–26.0 mm in males).

In having black spots on flanks, the new species differs from *L.aerea*, *L.botsfordi*, *L.crorea*, *L.firthi*, *L.isos*, *L.pallida*, *L.petrops*, and *L.tuberosa*, all of which lacking black spots on flanks; by having rudimentary webbing on toes, the new species differs from *L.oshanensis*, *L.pallida* and *L.petrops*, all of which lacking webbing on toes, and from *L.pelodytoides*, which bears wide webbing on toes; by having narrow lateral fringes on toes, the new species differs from *L.aerea*, *L.firthi*, *L.isos*, *L.khasiorum*, *L.laui*, *L.liui*, *L.purpura*, *L.yunkaiensis*, and *L.yingjiangensis*, all of which having wide lateral frings on toes, from *L.fuliginosa*, which having weak lateral fringes on toes, and from *L.crocea*, *L.macrops*, *L.minima*, *L.oshanensis*, *L.pallida*, *L.pyrrhops*, *L.tuberosa*, and *L.ventripunctata*, all of which lacking lateral fringes on toes; by having dorsal surface shagreened and granular, lacking enlarge tubercles or warts, the new species differs from *L.fuliginosa* (dorsum smooth with fine tubercles), *L.laui* (dorsum with round granular tubercle, lacking skin ridges), *L.liui* (dorsum with round tubercles), *L.macrops* (dorsum roughly granular with large tubercles), *L.minima* (dorsum smooth), *L.pelodytoides* (dorsum with small, smooth warts), *L.tuberosa* (dorsum highly tuberculate), *L.yunkaiensis* (dorsum with raised warts), and *L.wuhuangmontis* (dorsum rough with conical tubercles); by having ventral surface grey purple with distinct nebulous greyish speckling on chest and ventrolateral flanks, the new species differs from *L.botsfordi* and *L.pyrrhops*, (ventral reddish brown with white speckling), *L.khasiorum* (ventral creamy white), *L.macrops* (ventral Greyish-violet with white speckling), *L.nokrekensis* (ventral creamy white), *L.puhoatensis* (ventral reddish brown with white dusting), *L.purpura* (ventral dull white with indistinct grey dusting), *L.tuberosa* (ventral white with small grey spots/streaks), *L.ventripunctata* (chest and belly with large dark brown spots), *L.wuhuangmontis* (ventral greyish white), *L.yunkaiensis* (belly pink with speckling), and *L.yingjiangensis* (ventral creamy white); by having tiny spines on surface of chest extending to anterior region of abdomen in males during breeding season, the new species differs from all male specimens collected in breeding season of *L.liui*, *L.oshanensis*, *L.yunkaiensis* and *L.wuhuangmontis*, all of which lacking such spines.

##### Description of holotype.

Adult male. Body size small, SVL in 29.6 mm. Head length slightly larger than head width, HDL/HDW 1.05; snout slightly protruding, projecting slightly beyond margin of the lower jaw; nostril closer to snout than eye; canthus rostralis gently rounded; loreal region slightly concave; interorbital space flat, internarial distance larger than interorbital distance, IND/IOD 1.09; pineal ocellus absent; vertical pupil; snout length larger than eye diameter, SNT/EYE 1.14; tympanum distinct, rounded, and slightly concave, diameter smaller than that of the eye and larger than tympanum-eye distance, TMP/EYE 0.54 and TEY/TMP 0.68; upper margin of tympanum incontact with supratympanic ridge; distinct black supratympanic line present; vomerine teeth absent; vocal sac openings slit-like, paired, located posterolaterally on floor of mouth in close proximity to the margins of the mandible; tongue deeply notched behind; supratympanic ridge distinct, extending from posterior corner of eye to supra-axillary gland.

Tips of fingers rounded, slightly swollen; relative finger lengths I = II = IV < III; nuptial pad absent; subarticular tubercles absent; a large, rounded inner palmar tubercle distinctly separated from small, round outer palmar tubercle; absence of webbing and lateral fringes on fingers. Tips of toes like fingers; relative toe length I < II < V < III < IV; subarticular tubercles absent; distinct dermal ridges present under the 3^rd^ to 5^th^ toes, not interrupted; large, oval inner metatarsal tubercle present, outer metatarsal tubercle absent; toes webbing rudimentary; narrow lateral fringes present on all toes. Tibia 45% of snout-vent length; tibiotarsal articulation reaches to middle of eye; heels just meeting each other when thighs are appressed at right angles with respect to body.

Dorsal surface shagreened and granular, lacking enlarge tubercles or warts, some of the granules forming short longitudinal folds; ventral skin smooth; dense tiny conical spines present on surface of chest and extending to anterior region of abdomen; pectoral gland and femoral gland oval; pectoral glands greater than tips of fingers and femoral glands; femoral gland situated on posteroventral surface of thigh, closer to knee than to vent; supra-axillary gland raised. Ventrolateral gland distinctly visible, forming an incomplete line.

##### Measurements of holotype (in mm).

SVL 29.6, HDL 10.2, HDW 9.7, SNT 4.0, EYE 3.5, IOD 3.2, IND 3.5, TMP 1.9, TEY 1.3, TIB 13. 3, ML 7.7, PL 12.7, LAHL 13.8, HLL 42.7.

##### Coloration of holotype in life.

Dorsum dark purple brown grounding, with small light orange granules, distinct darker brown markings and rounded spots and scattered with irregular light orange pigmentations. A dark brown V-shaped pattern between anterior corner of eyes, in connected to the dark brown W-shaped marking on interorbital region, and the W-shaped marking in connected to the other W-shaped marking between axillae. Tympanum brown. A dark brown vertical bar under the eye; transverse dark brown bars on dorsal surface of limbs; distinct dark brown blotches on flanks from groin to axilla, longitudinally in two rows; elbow and upper arms with dark bars and distinct coppery orange coloration; fingers and toes with distinct dark bars.

Ventral surface grey purple, with distinct nebulous greyish speckling scattered with white spots on chest and ventrolateral flanks. Supra-axillary gland coppery orange with dark brown speckling; femoral, pectoral and ventrolateral glands greyish white. Iris bicolored, coppery orange on upper half and silver on lower half.

##### Coloration of holotype in preservative.

Dorsum of body and limbs dark brown; transverse bars on limbs become more distinct; dark brown patterns, markings and spots on back become indistinct, orange pigmentations become greyish white. Ventral surface of body and limbs greyish white, nebulous speckling on chest and flanks balck brown. Supra-axillary, femoral, pectoral and ventrolateral glands greyish white.

##### Variations.

**Figure 8.**
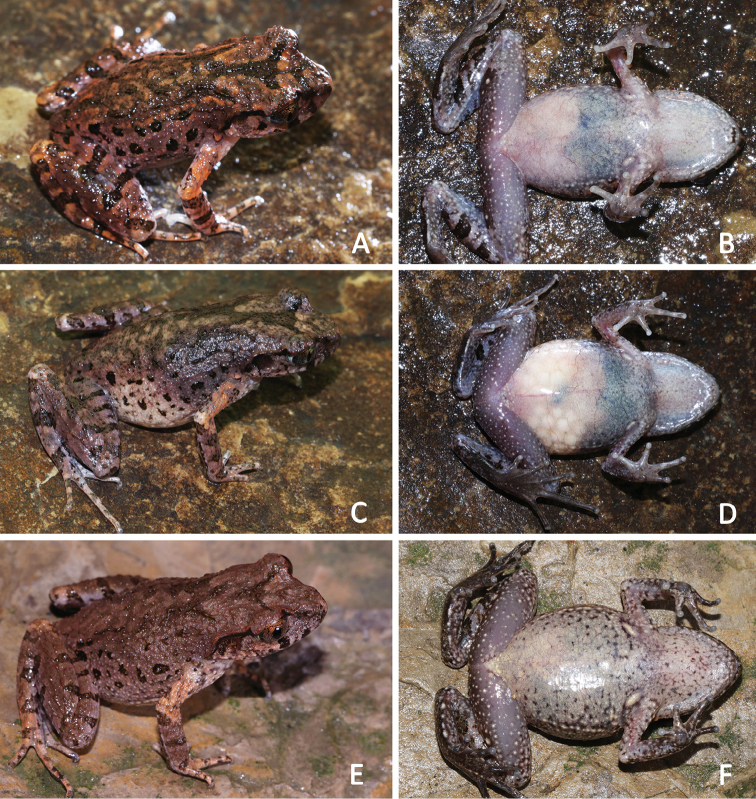
General aspect in life of the female paratypes of *Leptobrachellapurpuraventra* sp. nov. **A, B**SYS a007304 **C, D**SYS a007305 **E, F**SYS a007278.

Measurements and body proportions were listed in Table 7. All paratypes match the overall characters of the holotype except that: coloration of dorsum dark purple brown in the holotype SYS a007284 (vs. grey purple brown in paratypes SYS a007300 (Figure 7E), 7303, 7305 (Figure 8C), 7306; purple brown in paratypes SYS a007278 (Figure 8E), 7279, 7282, 7283 (Figure 7F), 7304 (Figure 8A)); heels just meeting (vs. heels slightly overlapping in paratypes SYS a007300, 7302); W-shaped marking on interorbital region in connected to the other W-shaped marking between axillae in the holotype (vs. such markings not in connected with each other in paratypes SYS a007278, 7282); a V-shaped pattern between anterior corner of eyes in the holotype (vs. a dark brown inverted triangular pattern between anterior corner of eyes instead in the paratype SYS a007300); relatively smaller black spots on flanks (vs. black spots distinctly large in paratypes SYS a007300–7301, 7304, 7306); ventral surface without black spots in the holotype (vs. presence of irregular black spots in paratype SYS a007278 (Figure 8F)).

**Table 7.** Measurements (minimum–maximum (mean ± SD); in mm), and body proportions of *Leptobrachellapurpuraventra* sp. nov.: population A from Wujing Nature Reserve in Bijie City, population B from Baimashan Forest Station of Zhaozishan Nature Reserve.

Population	A		B		A + B	
SEX	Males	Female	Males	Females	Males	Females
	(n = 7)	(n = 1)	(n = 4)	(n = 3)	(n = 11)	(n = 4)
**SVL**	27.3–29.6	35.3	28.3–29.8	33.0–34.5	**27.3–29.8**	**33.0–35.3**
	(28.6 ± 0.7)		(29.3 ± 0.6)	(33.5 ± 0.7)	**(28.9 ± 0.8)**	**(34.0 ± 1.0)**
**HDL**	9.6–10.2	12	9.7–10.3	11.0–11.7	**9.6–10.3**	**11.0–12.0**
	(9.9 ± 0.2)		(10.1 ± 0.2)	(11.3 ± 0.3)	**(10.0 ± 0.2)**	**(11.5 ± 0.4)**
**HDW**	9.3–9.7	11.5	9.6–9.8	10.5–11.3	**9.3–9.8**	**10.5–11.5**
	(9.5 ± 0.1)		(9.8 ± 0.1)	(10.9 ± 0.3)	**(9.6 ± 0.2)**	**(11.1 ± 0.4)**
**SNT**	3.5–4.0	4.6	3.8–4.1	4.2–4.4	**3.5–4.1**	**4.2–4.6**
	(3.8 ± 0.1)		(4.0 ± 0.1)	(4.3 ± 0.1)	**(3.8 ± 0.2)**	**(4.4 ± 0.1)**
**EYE**	3.1–3.5	3.8	3.3–3.6	3.7–3.9	**3.1–3.6**	**3.7–3.9**
	(3.3 ± 0.2)		(3.5 ± 0.1)	(3.8 ± 0.1)	**(3.4 ± 0.2)**	**(3.8 ± 0.1)**
**IOD**	2.6–3.2	3.5	3.0–3.2	3.2–3.3	**2.6–3.2**	**3.2–3.5**
	(2.9 ± 0.2)		(3.1 ± 0.1)	(3.2 ± 0.1)	**(2.9 ± 0.2)**	**(3.3 ± 0.1)**
**IND**	2.7–3.5	3.6	3.2–3.3	3.3–3.5	**2.7–3.5**	**3.3–3.6**
	(3.0 ± 0.3)		(3.0 ± 0.1)	(3.4 ± 0.1)	**(3.1 ± 0.2)**	**(3.5 ± 0.1)**
**TMP**	1.7–1.9	2.1	1.8–1.9	2.0–2.1	**1.7–1.9**	**2.0–2.1**
	(1.8 ± 0.1)		(1.8 ± 0.1)	(2.0 ± 0.1)	**(1.8 ± 0.1)**	**(2.1 ± 0.1)**
**TEY**	1.2–1.3	1.3	1.1–1.2	1.2–1.3	**1.1–1.3**	**1.2–1.3**
	(1.3 ± 0.1)		(1.1 ± 0.1)	(1.2 ± 0.1)	**(1.2 ± 0.1)**	**(1.3 ± 0.1)**
**TIB**	12.5–13.3	15.5	13.2–14.0	14.6–15.4	**12.5–14.0**	**14.6–15.5**
	(12.8 ± 0.3)		(13.6 ± 0.4)	(15.0 ± 0.3)	**(13.1 ± 0.5)**	**(15.2 ± 0.4)**
**ML**	7.0–7.7	7.8	7.5–7.6	7.7–8.0	**7.0–7.7**	**7.7–8.0**
	(7.3 ± 0.2)		(7.5 ± 0.1)	(7.9 ± 0.1)	**(7.4 ± 0.2)**	**(7.9 ± 0.1)**
**PL**	12.1–12.7	14.8	12.6–13.2	13.7–14.7	**12.1–13.2**	**13.7–14.8**
	(12.4 ± 0.2)		(13.0 ± 0.2)	(14.2 ± 0.4)	**(12.6 ± 0.4)**	**(14.4 ± 0.4)**
**LAHL**	12.6–13.8	15.5	13.4–14.0	14.7–15.7	**12.6–14.0**	**14.7–15.7**
	(13.2 ± 0.4)		(13.6 ± 0.2)	(15.1 ± 0.4)	**(13.3 ± 0.4)**	**(15.2 ± 0.4)**
**HLL**	39.0–42.7	47.8	40.1–44.6	46.0–47.3	**39.0–44.6**	**46.0–47.8**
	(40.4 ± 1.4)		(43.2 ± 1.8)	(46.8 ± 0.6)	**(41.4 ± 2.1)**	**(47.0 ± 0.7)**
**HDL/HDW**	1.01–1.05	1.04	1.01–1.06	1.04–1.05	**1.01–1.06**	**1.04–1.05**
	(1.04 ± 0.01)		(1.04 ± 0.02)	(1.04 ± 0.01)	**(1.04 ± 0.02)**	**(1.04 ± 0.01)**
**HDL/SVL**	0.33–0.35	0.34	0.34–0.35	0.33–0.34	**0.33–0.35**	**0.33–0.34**
	(0.34 ± 0.02)		(0.34 ± 0.01)	(0.34 ± 0.01)	**(0.34 ± 0.01)**	**(0.34 ± 0.01)**
**SNT/HDL**	0.36–0.39	0.38	0.39–0.40	0.38	**0.36–0.40**	**0.38**
	(0.38 ± 0.01)		(0.39 ± 0.01)		**(0.39 ± 0.01)**	
**SNT/EYE**	1.12–1.19	1.21	1.11–1.15	1.11–1.16	**1.11–1.19**	**1.11–1.21**
	(1.14 ± 0.02)		(1.14 ± 0.02)	(1.13 ± 0.02)	**(1.14 ± 0.02)**	**(1.15 ± 0.04)**
**EYE/TMP**	1.82–1.89	1.81	1.83–1.94	1.85–1.90	**1.82–1.94**	**1.81–1.90**
	(1.86 ± 0.03)		(1.89 ± 0.04)	(1.87 ± 0.02)	**(1.87 ± 0.04)**	**(1.85 ± 0.03)**
**TMP/EYE**	0.53–0.55	0.55	0.51–0.55	0.53–0.54	**0.51–0.55**	**0.53–0.55**
	(0.54 ± 0.01)		(0.53 ± 0.01)	(0.53 ± 0.01)	**(0.53 ± 0.01)**	**(0.54 ± 0.01)**
**IND/IOD**	1.03–1.09	1.03	1.03–1.10	1.03–1.06	**1.03–1.10**	**1.03–1.06**
	(1.06 ± 0.02)		(1.07 ± 0.03)	(1.05 ± 0.01)	**(1.07 ± 0.03)**	**(1.05 ± 0.02)**
**TEY/TMP**	0.67–0.76	0.62	0.61–0.67	0.60–0.62	**0.61–0.76**	**0.60–0.62**
	(0.71 ± 0.03)		(0.64 ± 0.02)	(0.61 ± 0.01)	**(0.68 ± 0.04)**	**(0.61 ± 0.01)**
**TIB/SVL**	0.44–0.46	0.44	0.45–0.47	0.44–0.46	**0.44–0.47**	**0.44–0.46**
	(0.45 ± 0.01)		(0.47 ± 0.01)	(0.45 ± 0.01)	**(0.45 ± 0.01)**	**(0.45 ± 0.01)**
**LAHL/SVL**	0.45–0.47	0.44	0.45–0.47	0.44–0.46	**0.45–0.47**	**0.44–0.46**
	(0.46 ± 0.01)		(0.46 ± 0.01)	(0.45 ± 0.01)	**(0.46 ± 0.01)**	**(0.45 ± 0.01)**
**HLL/SVL**	1.36–1.46	1.35	1.42–1.51	1.37–1.42	**1.36–1.51**	**1.35–1.42**
	(1.41 ± 0.03)		(1.47 ± 0.04)	(1.39 ± 0.02)	**(1.43 ± 0.05)**	**(1.38 ± 0.02)**
**TIB/HLL**	0.31–0.32	0.32	0.31–0.33	0.32–0.33	**0.31–0.33**	**0.32–0.33**
	(0.31 ± 0.01)		(0.32 ± 0.01)	(0.32 ± 0.01)	**(0.32 ± 0.01)**	**(0.32 ± 0.01)**

##### Etymology.

The specific epithet *pupura* is given as a noun in apposition and means “purple color”, and *ventra*, is given as a noun in apposition and means “ventral”, in reference to the purple coloration of ventral of the new species. For the common name, we suggest “Purple-bellied Leaf Litter Toad”, and for the Chinese name “Zi Fu Zhang Tu Chan (紫腹掌突蟾)”.

##### Distribution and habits.

**Figure 9.**
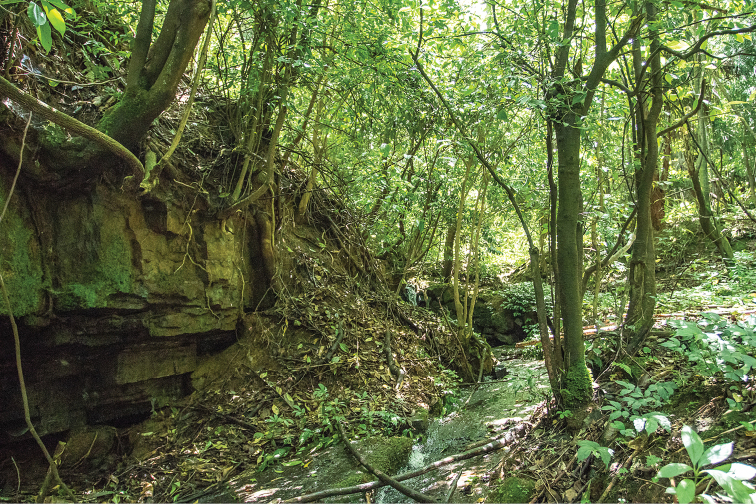
The habitat of *Leptobrachellapurpuraventra* sp. nov. in Baimashan Forest Station of Zhaozishan Nature Reserve in Guizhou Province.

**Figure 10.**
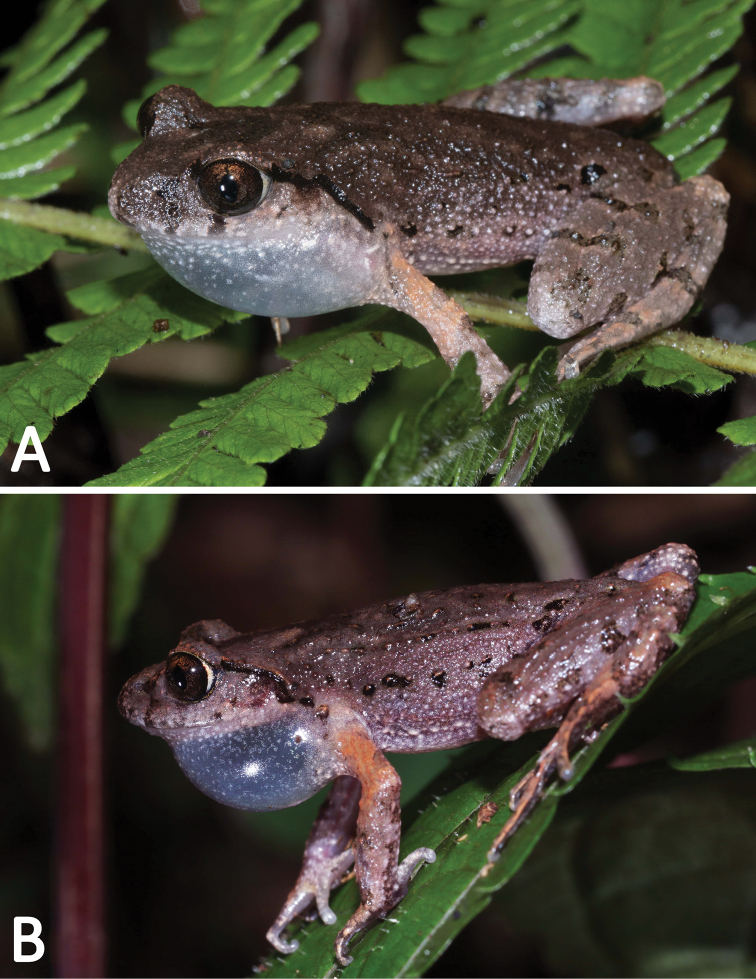
General aspect observed in the field of **A***Leptobrachellabijie* sp. nov. and **B***L.purpuraventra* sp. nov., showing a single vocal sac and different skin colors of the two new species.

Currently, *Leptobrachellapurpuraventra* sp. nov. is known from its type locality Jinjiazhai Village in Wujing Nature Reserve, Chahe County, and Baimashan Forest Station in Zhaozishan Nature Reserve, both in Qixingguan District, Bijie City, Guizhou Province, China (Figure 1). The new species was found along a clear-water rocky stream (ca. 3 m in width and ca. 10–20 cm in depth) surrounded by a broad-leaved forest in karst landforms (Figure 9, 1600–1900 m a.s.l.). From 2 July to 4 July in 2018 at 21:00–23:50 P.M., a large number of males were found calling on leaves of plants (Figure 10B), and some were found calling perching on the rocks or under rocks by the side of the stream.

## Discussion

The discoveries of *Leptobrachellabijie* sp. nov. and *L.purpuraventra* sp. nov. bring the total number of this genus to 73, with 16 of them recorded in China (Fei et al. 2012; Frost 2017; Wang et al. 2018). Before the descriptions of the two new species from northwestern Guizhou Province in this study, only *L.oshanensis* was recorded in northeastern and southern Guizhou Province, which further highlights the underestimated of the species diversity of the genus. Further investigation of the genus in adjacent regions is required.

Studies of the taxonomy and phylogeny of *Leptobrachella* were difficult to perform because of the morphological conservativeness of the species (for example, the two new species appeared very similar morphologically in the field (Figure 10)), which likely to hinder our understanding of these cryptic species (Ohler et al. 2010; Sung et al. 2014; Wang et al. 2018).

*Leptobrachellabijie* sp. nov. and *L.purpuraventra* sp. nov. were both found in Zhaozishan Nature Reserve, only approximately seven kilometers apart, straight-line distance, but they possessed a significant genetic divergence (*p*=3.9–4.2%). This compares to the two populations of *L.purpuraventra* sp. nov. from Zhaozishan Nature Reserve and Wujing Nature Reserve, which were approximately 65 kilometers apart, but displayed almost no genetic divergence. Without phylogenetic, morphological, and acoustic analyses, it would be difficult to determine the taxonomic status of these two species. Thus, specimen, acoustic data, and tissue sample collection play important roles in discovering the high species diversity of the genus *Leptobrachella*.

*Leptobrachellabijie* sp. nov. and *L.purpuraventra* sp. nov. were found along clear-water rocky streams, and such environments are very limited in the karst landforms. At present, little is known about the ecology and behavior of the two new species, however, the known habitat of the two new species is under threat of degradation, particularly as a result of grazing. Thus, further research on the true distribution, population size and trends, and conservation actions required, are urgently needed.

## Supplementary Material

XML Treatment for Leptobrachella bijie

XML Treatment for Leptobrachella purpuraventra

## Appendix 1

**Specimens examined**

*Leptobrachellaalpinus* (n = 6): China: Yunnan Province: Jingdong County: Mt. Wuliang: CIB 24353 (Holotype), CIB 24354; SYS a 003927.

*Leptobrachellalaui* (n = 26): China: Hong Kong: SYS a002057 (Holotype), SYS a002058; China: Guangdong Province: Shenzhen City: SYSa 001505–1507, 1515–1521, 3471–3472, 5644–5645.

*Leptobrachellaliui* (n = 18): China: Fujian Province: Mt. Wuyi: CIB 24355 (Holotype), CIB 24356, SYS a001571–1578, 1595–1599, 2478–2479, 5925–5826.

*Leptobrachellaoshanensis* (n = 2): China: Sichuan Province: Meishan City: Mt. Emei: SYS a001829–1830.

*Leptobrachellatengchongensis* (n = 6): China: Yunnan Province: Baoshan City: Mt. Gaoligong: SYS a004600 (Holotype), 4596–4599, 4601–4602.

*Leptobrachellawuhuangmontis* (n = 12): China: Guangxi Province: Pubei County: Mt. Wuhuang: SYS a003500/CIB107274, SYS a000578, 0580–0581, 3485–3489, 3499, 3504–3506.

*Leptobrachellayunkaiensis* (n = 8): China: Guangdong Province: Maoming City: Dawuling Forest Station: SYS a004664/CIB107272, SYS a004663, 4665–4669, 4690.
